# Hybridization may aid evolutionary rescue of an endangered East African passerine

**DOI:** 10.1111/eva.13440

**Published:** 2022-07-04

**Authors:** Daniel Vedder, Luc Lens, Claudia A. Martin, Petri Pellikka, Hari Adhikari, Janne Heiskanen, Jan O. Engler, Juliano Sarmento Cabral

**Affiliations:** ^1^ Ecosystem Modelling Group, Center for Computational and Theoretical Biology University of Würzburg Würzburg Germany; ^2^ Department of Ecosystem Services Helmholtz Centre for Environmental Research – UFZ Leipzig Germany; ^3^ Institute of Biodiversity Friedrich Schiller University Jena Jena Germany; ^4^ German Centre for Integrative Biodiversity Research (iDiv) Halle‐Jena‐Leipzig Leipzig Germany; ^5^ Terrestrial Ecology Unit, Biology Department Ghent University Ghent Belgium; ^6^ Department of Geosciences and Geography University of Helsinki Helsinki Finland; ^7^ State Key Laboratory for Information Engineering in Surveying, Mapping and Remote Sensing Wuhan University Wuhan China; ^8^ Landscape Research, Department of Geography Ghent University Ghent Belgium; ^9^ Computational Landscape Ecology Technische Universität Dresden Dresden Germany

**Keywords:** evolutionary rescue, habitat change, individual‐based model, introgressive hybridization, Taita Hills, *Zosterops silvanus*

## Abstract

Introgressive hybridization is a process that enables gene flow across species barriers through the backcrossing of hybrids into a parent population. This may make genetic material, potentially including relevant environmental adaptations, rapidly available in a gene pool. Consequently, it has been postulated to be an important mechanism for enabling evolutionary rescue, that is the recovery of threatened populations through rapid evolutionary adaptation to novel environments. However, predicting the likelihood of such evolutionary rescue for individual species remains challenging. Here, we use the example of *Zosterops silvanus*, an endangered East African highland bird species suffering from severe habitat loss and fragmentation, to investigate whether hybridization with its congener *Zosterops flavilateralis* might enable evolutionary rescue of its Taita Hills population. To do so, we employ an empirically parameterized individual‐based model to simulate the species' behaviour, physiology and genetics. We test the population's response to different assumptions of mating behaviour and multiple scenarios of habitat change. We show that as long as hybridization does take place, evolutionary rescue of *Z. silvanus* is likely. Intermediate hybridization rates enable the greatest long‐term population growth, due to trade‐offs between adaptive and maladaptive introgressed alleles. Habitat change did not have a strong effect on population growth rates, as *Z. silvanus* is a strong disperser and landscape configuration is therefore not the limiting factor for hybridization. Our results show that targeted gene flow may be a promising avenue to help accelerate the adaptation of endangered species to novel environments, and demonstrate how to combine empirical research and mechanistic modelling to deliver species‐specific predictions for conservation planning.

## 1 INTRODUCTION

Hybridization is a fiercely contentious topic in conservation biology. It has often been seen negatively, as a process that threatens the genetic integrity of species (Allendorf et al., 2001), and that may even lead to ‘extinction by hybridisation’ through phenomena such as outbreeding depressions (Rhymer & Simberloff, 1996). On the contrary, the very concept of genetic integrity has been strongly critiqued (Rohwer & Marris, 2015), and it is well‐known that introgressive hybridization, that is the backcrossing of hybrids into a parent population, can be a mechanism for interspecific gene flow (Uecker et al., 2015). In this context, it has been suggested to be an important enabler of evolutionary rescue (Baskett & Gomulkiewicz, 2011).

The concept of evolutionary rescue describes the rapid adaptation and subsequent population recovery of a species threatened by environmental change and has been much discussed in recent years (e.g. De Meester et al., 2018; Gonzalez et al., 2013; Tomasini & Peischl, 2020). Although there are some observed instances of species that may be considered to have undergone evolutionary rescue, many authors doubt its applicability to the majority of threatened species (De Meester et al., 2018; Vander Wal et al., 2013). However, introgressive hybridization may make genetic variation available much more rapidly than mutations could, and thus overcome the normal barriers to evolutionary rescue (Baskett & Gomulkiewicz, 2011; Stelkens et al., 2014). Significantly, this has been observed between congeneric species in the wild, for instance affecting winter coat polymorphism in snowshoe hares (Jones et al., 2018) and pollution resistance in killifish (Oziolor et al., 2019). In fact, human‐mediated hybridization is even being discussed as a species conservation measure (Hamilton & Miller, 2016).

Predicting whether or not a given species is likely to undergo evolutionary rescue can thus be relevant for conservation management (Derry et al., 2019; Pierson et al., 2015). It remains a major research challenge though, as doing so requires extensive empirical knowledge of the species' ecology and the use of mechanistic models (Gomulkiewicz & Shaw, 2013). Consequently, it is only rarely done (e.g. Gienapp et al., 2013; Razgour et al., 2019), and most studies of evolutionary rescue restrict themselves to abstract theoretical models or laboratory‐based experiments. Here we show how an empirically parameterized, genetically explicit individual‐based model can be used to investigate the likelihood of evolutionary rescue by introgressive hybridization in the case of the endangered East African passerine bird species *Zosterops silvanus*.

*Zosterops silvanus* is a highland species endemic to the Taita Hills and Mt Kasigau in southern Kenya, which form part of the highly diverse Eastern Arc Mountains. The species is a habitat specialist, breeding in montane cloud forests. Its small range and the large‐scale deforestation of the Taita Hills means that it is considered endangered, with the last published census estimating a global population of 7100 individuals (Mulwa et al., 2007), and probably far fewer remaining today (del Hoyo et al., 2020). Despite systematic conservation efforts in the Taita Hills (Githiru et al., 2011), the future development of its remaining montane forest fragments remains uncertain (Teucher et al., 2020), posing a strong threat to *Z. silvanus* and other Taita endemics.

As *Z. silvanus* is often observed in mixed feeding flocks with its lowland congener *Zosterops flavilateralis*, we hypothesize that hybridization between the two species is possible, given the many documented cases of hybridization in other *Zosterops* species (McCarthy, 2006). Recent genetic clustering analyses on polymorphic microsatellite markers further showed a much higher degree of variability in *Z. silvanus* populations as compared with other montane *Zosterops* lineages (Habel et al., 2014), which may be attributable to admixture with a lowland species such as *Z. flavilateralis*. Since *Z. flavilateralis* breeds in more open habitats rather than the montane forests, we wanted to know whether hybridization between the two species might affect the ability of *Z. silvanus* to rapidly adapt to new habitats and increase its population size.

To investigate this, we used GeMM, a spatially and genetically explicit individual‐based model that has previously been used for eco‐evolutionary (Leidinger et al., 2021) and conservation‐oriented (Vedder, Leidinger, & Cabral, 2021) studies. We adapted and parameterized this model to fit the known behaviour and life‐history of the two *Zosterops* species. To provide a realistic high‐resolution model landscape of the Taita Hills, we used results from mapping of land cover and above‐ground carbon in various land‐use types derived from airborne laser scanner data and satellite imagery (Adhikari et al., 2017; Pellikka et al., 2018).

Our study was driven by two questions. As we do not yet have any empirical estimates for hybridization rates between *Z. silvanus* and *Z. flavilateralis*, Question 1 was to explore how different assumptions of mating behaviour (i.e. different hybridization propensities) would influence the possibility of evolutionary rescue. Based on previous studies, we expected low‐to‐intermediate hybridization rates to provide the best conditions for evolutionary rescue (Baskett & Gomulkiewicz, 2011). For Question 2, we investigated how spatial landscape effects might affect hybridization rates and, consequently, evolutionary rescue. Specifically, we wanted to know what effects planned conservation work and ongoing habitat destruction in the Taita Hills are likely to have on *Z. silvanus* population development, particularly with regard to hybridization‐mediated evolutionary rescue. We expected that increased fragmentation, characterized by lower habitat connectivity and smaller overall habitat area, would increase hybridization rates, whereas decreased fragmentation would decrease hybridization rates (cf. Seifert et al., 2010).

## 2 METHODS

### 2.1 Model description

For this study, we adapted the GeMM model of Leidinger et al. (2021) to reflect the biology of the *Zosterops* species in the Taita Hills. GeMM is implemented in Julia with the Distributions.jl package (Bezanson et al., 2017; Lin et al., 2021), while data preparation and analysis for this study was carried out in R 4.0.4 with the tidyverse packages (R Core Team, 2021; Wickham et al., 2019). Software development and verification followed best practice as described in Vedder, Ankenbrand, and Cabral (2021). The full model description following the ODD protocol (Grimm et al., 2010) can be found in the Supplementary Materials. The source code and accompanying documentation are freely available at https://github.com/CCTB‐Ecomods/gemm.

The model map consists of a grid of one‐hectare patches, covering 962 km of the landscape of the Taita Hills (Figure S2). (Terminology note: throughout, we use ‘patch’ to refer to a single one‐hectare grid cell in the model, and ‘fragment’ to refer to a contiguous area of montane forest habitat in the landscape.) Each patch is primarily characterized by its mass of above‐ground carbon (AGC, in Mg C ha^−1^). These data were collected in 2013–2015 using a combination of airborne laser scanning and field measurements (Adhikari et al., 2017), and allow a differentiation of local habitat types such as montane forest, exotic forest, woodland and cropland (Pellikka et al., 2018). As the observed population densities of *Zosterops* vary between habitat types (Mulwa et al., 2007; J. Engler, unpublished data), the AGC values were also used to calculate the number of adult birds that may breed in a patch (i.e. patch‐specific carrying capacities).

The fundamental entities of the model are individual birds (Figure S1). Each bird has a diploid genome comprised of multiple chromosomes, each of which by default contains one gene that codes for one trait. For this study, the relevant traits are those pertaining to the birds' habitat adaptation (i.e. AGC optimum and AGC tolerance) and dispersal distance (i.e. mean and shape parameters of the dispersal kernel). Each bird also has a species label and a sex, and may acquire a mating partner.

During initialization, birds are created with species‐specific trait values for either *Z. silvanus* or *Z. flavilateralis* (Table S2). *Z. silvanus* was assigned an AGC range (i.e. AGC optimum ± AGC tolerance) of 90–270 Mg C ha^−1^, and *Z. flavilateralis* a range of 3–97 Mg C ha^−1^. This reflects the AGC values of montane forest habitat and all other vegetation types, respectively (Pellikka et al., 2018). Habitat patches whose AGC values fall within the AGC tolerance range of a species are populated with a random number of new bird individuals of that species, while assuring that the total patch community size does not exceed its carrying capacity.

During a simulation run, each model iteration represents one year. In every iteration, all individual birds undergo three main life‐history processes: survival, reproduction and dispersal.

#### 2.1.1 Survival

Survival is simulated as an annual, fixed, density‐independent survival probability for each bird, based on the known life‐expectancy of approximately 8 years for related *Zosterops* species (Bird et al., 2020).

#### 2.1.2 Reproduction

Breeding pairs mate every year, producing zero, one or two offspring. This range was chosen to reflect *Zosterops* clutch sizes, breeding attempts and juvenile mortality (Abdar, 2014; del Hoyo et al., 2020; J. Engler, unpublished data). During reproduction, the parents' genomes undergo meiosis, thus enabling genetic recombination of the offsprings' genomes. The offspring's phenotype for each trait is calculated by averaging the values of its paternal and maternal alleles for that trait. If the offspring is a hybrid, it is assigned to the species of the parent to which it is phenotypically more similar. Breeding pairs stay faithful for life and always remain in their patch. If one partner dies, the other may form a new breeding pair with a new bird that disperses into its patch.

#### 2.1.3 Dispersal

Our dispersal algorithm represents an adapted version of the Stochastic Movement Simulator, developed by Palmer et al. (2011) to estimate habitat connectivity in heterogeneous landscapes. This algorithm has previously been tested against GPS‐tracking data of other forest bird species in the Taita Hills (Aben et al., 2014). Juvenile birds leave their native patch and repeatedly move to the neighbouring patch whose habitat is closest to their AGC optimum value, until they either find a suitable patch to settle or exceed their maximum dispersal distance and die. The maximum dispersal distance is calculated using a dispersal kernel with parameters based on a capture‐recapture study of *Z. silvanus* (Lens et al., 2002). A patch is suitable if it is within the bird's AGC tolerance range, and either has at least two free slots in its carrying capacity so the dispersing bird can stake out a territory for a new breeding pair, or there is a resident, partner‐less bird of the opposite sex with which the dispersing bird can form a new breeding pair. If there are birds of both species in the same patch, dispersing birds will always try to mate with a conspecific. If there is no conspecific mate available, the hybridization propensity parameter defines the probability with which a bird may also accept an extraspecific mate, that is hybridize.

### 2.2 Experimental design

**FIGURE 1:**
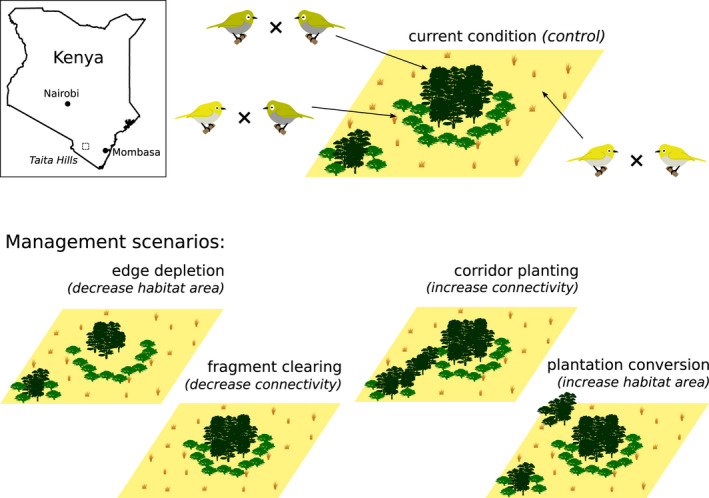
Conceptual illustration of the experimental set‐up. *Zosterops silvanus* (green birds) occur in montane forest habitats, whereas *Zosterops flavilateralis* (yellow birds) occur in the more open habitats. They can hybridize in intermediate habitats with a given probability, if there are no available conspecific mates. For the simulation experiments, we varied either the hybridization propensity or the model landscapes. Note that this figure is for illustration purposes, see the main text for a complete study description and Figure S2 for a map of the actual landscape. Inset shows the location of the Taita Hills in Kenya

To answer our two study questions, we conducted two separate simulation experiments to investigate the likely population development of *Z. silvanus* under different scenarios of hybridization propensity and habitat change (Figure 1). In both experiments and for all scenarios, we simulated 50 replicates over 300 years. We chose this duration because we were interested in medium‐term population dynamics that could show eco‐evolutionary responses while still being within a time frame that is relevant for conservation. Also, our results show that this duration was sufficient for population variables to come close to quasi‐stationary equilibrium levels (cf. Figure S3).

In the first simulation experiment (for Question 1), we used the habitat map from 2015 to represent current environmental conditions. In this setting, we set the hybridization propensity to either 0%, 1%, 5%, 10%, 50%, or 100%. As the actual hybridization propensity of *Z. silvanus* and *Z. flavilateralis* is not known, we chose this range of values to reflect different hypothetical assumptions about the species' mating behaviour. We note that there is a wide spread of estimates for hybridization rates in birds (Justyn et al., 2020; Ottenburghs & Slager, 2020), as these are very hard to measure empirically (Justen et al., 2021; Minor et al., 2022). Our best estimate is that a hybridization propensity between 1% and 10% is likely to produce realistic hybridization rates within the contact zones in the model, but as this is associated with large uncertainties, we wanted to test a wide range of values.

In the second simulation experiment (for Question 2), we explored the effects of possible habitat changes on hybridization frequency and population stability, while keeping the hybridization propensity constant at 1%. For this, we ran the model with five different scenario maps that increased or decreased habitat area or connectivity, based on the conservation history of the Taita Hills (Table S1).

The first scenario was the control or baseline scenario, using the 2015 map to simulate current conditions.

The second (‘edge depletion’) was a negative change scenario featuring habitat area loss by continued deforestation. This was simulated by the conversion of 149 ha of forest on the edges of the three major montane forest fragments into bushland/woodland (Teucher et al., 2020), achieved by assigning the ‘deforested’ patches an AGC value of 20 MgC ha^−1^ (the value associated with woodland; Pellikka et al., 2018).

The third (‘fragment clearing’) was another negative scenario, simulating the clearcut logging of four small montane forest fragments (total area: 58 ha), such as when clearing for agricultural land (cf. Pellikka et al., 2013). Compared with the second scenario, this third scenario preserved more habitat area, but had a greater impact on habitat connectivity through the removal of entire forest fragments.

The fourth (‘corridor planting’) was a positive change scenario that increased connectivity by modelling the corridor of indigenous trees that has recently been planted between three forest fragments (Wagura, 2018). The corridor was implemented as a one‐patch wide strip (85 ha total), with each patch set to an AGC value of 100 MgC ha^−1^ (cf. corridor modelling in Krug et al., 2010).

The fifth and final scenario (‘plantation conversion’) was again positive, implementing the recommendation of Githiru et al. (2011) to convert five exotic tree plantations to indigenous montane forest. This amounted to 294 ha that were set to 100 MgC ha^−1^. This scenario focussed mainly on adding large amounts of habitat area.

Additionally, we ran a number of exploratory simulation experiments to investigate the effects of various model assumptions on our main results. These included an experiment that disabled hybridization but allowed gene changes through mutations, a second experiment that tested the effects of genetic linkage (i.e. having several genes on the same chromosome) and a third that extended the simulation runtime to 1000 years. Furthermore, we repeated the habitat experiment with hybridization propensities of 0% and 10%. These additional results are reported in the Supplementary Materials, and we elaborate on the consequences of said assumptions in the Discussion.

### 2.3 Analyses

In the hybridization experiment for Question 1, we recorded the development of *Z. silvanus*' global population size over time as the key determinant of evolutionary rescue. We measured the extent of hybridization using the metric of population heterozygosity, that is the percentage of extraspecific chromosomes in the gene pool. To track the effects of hybridization on the population, we also plotted the temporal development of the populations' mean trait values for AGC optimum and AGC tolerance. To show how the species expands its habitat after hybridization, we plotted maps showing the spatial distribution of population densities at the end of the experiment.

In the habitat experiment for Question 2, we likewise recorded population size, heterozygosity, AGC optimum and AGC tolerance over time for each scenario. To see how landscape structure affects hybridization, we plotted maps of per‐patch heterozygosity levels. To quantify the effects of fragmentation more closely, we also calculated per‐scenario heterozygosity levels for a set of habitat fragments of various sizes that are known to be inhabited by *Z. silvanus* (Habel et al., 2014), and performed a linear regression of fragment heterozygosity against the log‐transformed fragment area.

As there is no distinction between ‘observed mean’ and ‘true mean’ in the results of simulation models, the fundamental assumption of significance tests is invalid. Therefore, we do not report *p*‐values of differences (White et al., 2014). Nevertheless, we provide means and distribution parameters of emergent results to help evaluate the differences among scenarios.

## 3 RESULTS

### 3.1 Hybridization experiment

**FIGURE 2:**
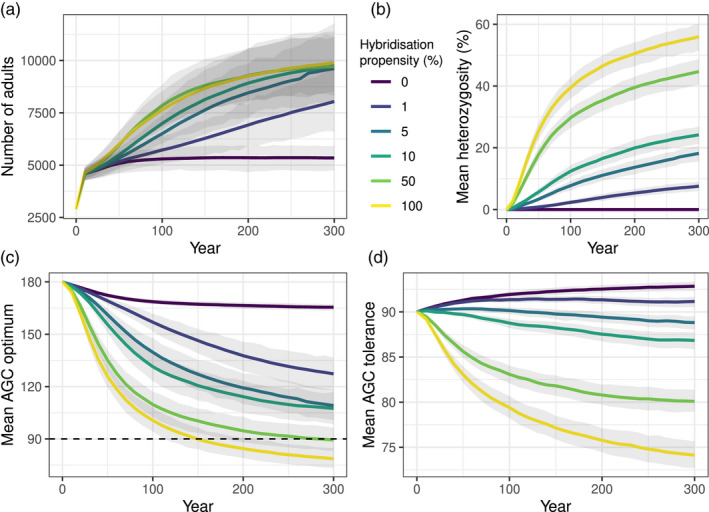
Development of key variables over 300 years in the hybridization experiment, differentiated by hybridization propensity. (a) Global number of adult *Z. silvanus* individuals. (b) Mean population heterozygosity of *Z. silvanus* (i.e. percentage of extraspecific chromosomes in the population gene pool). (c) Mean AGC optimum trait value of all *Z. silvanus* individuals. (d) Mean AGC tolerance trait value of all *Z. silvanus* individuals. Solid lines show the mean of 50 replicates, shaded areas are 95% confidence intervals. AGC: above‐ground carbon, in Mg C ha^−1^ (a proxy for habitat type, see main text). The dashed line in panel (c) denotes the boundary between montane forest habitats (AGC ≧ 90) and other habitat types (AGC < 90)

The population development of *Z. silvanus* was differentiated by the hybridization propensity. Whereas a hybridization propensity of 0% led to a very early and low population plateau, higher values led to increasingly faster population growth; although the final population sizes did not differ strongly for values of 5% and higher (Figure 2a).

Population heterozygosity likewise showed a logarithmic increase over time and was directly proportional to the hybridization propensity (Figure 2b). Population mean AGC optimum showed the inverse development to population heterozygosity, with strong drops corresponding to higher hybridization propensity settings (Figure 2c). Population mean AGC tolerance behaved similarly, but with the difference that in scenarios with a hybridization propensity of less than 5%, AGC tolerance rose slightly over time (Figure 2d).

**FIGURE 3:**
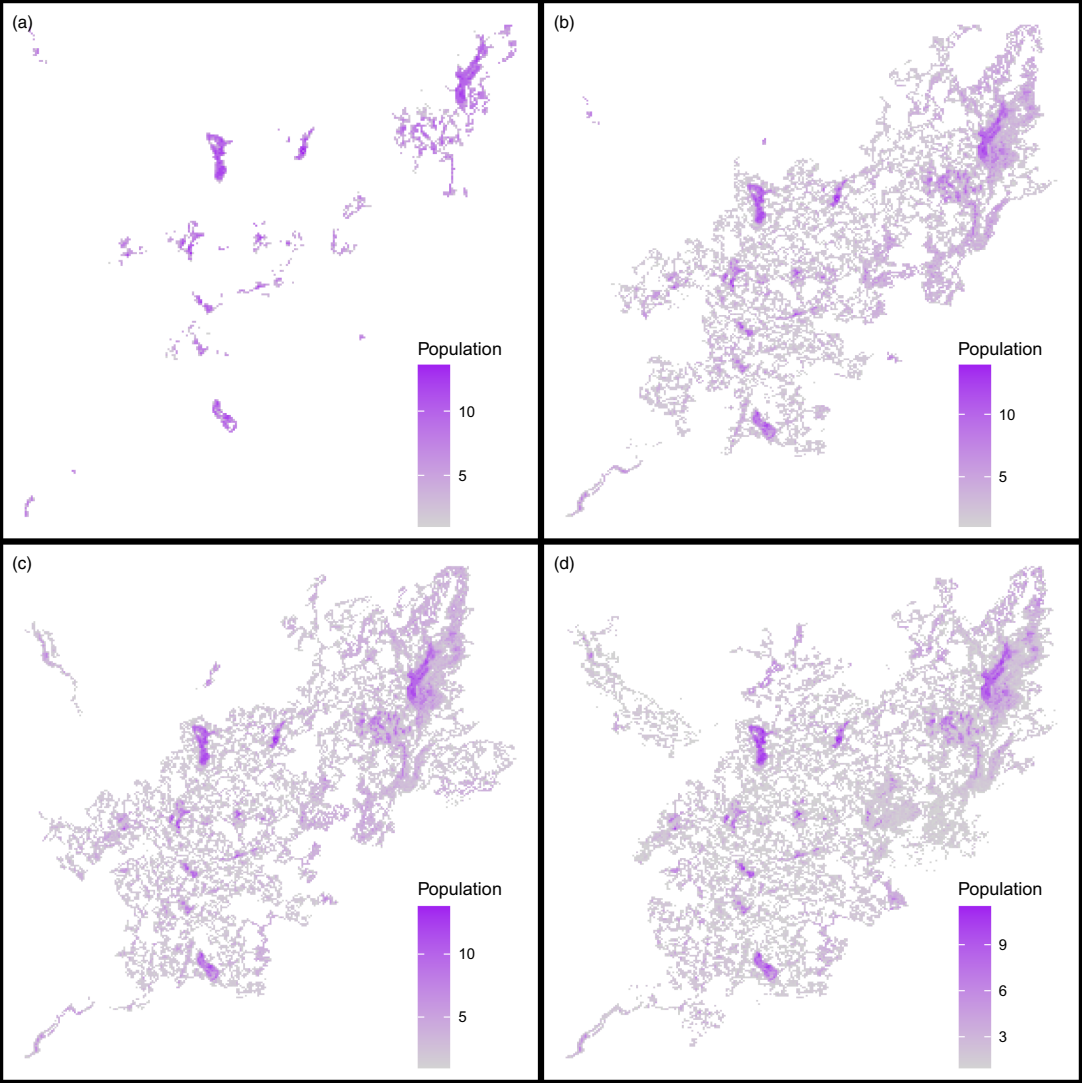
Spatial distribution of population density of *Z. silvanus* in the Taita Hills, Kenya, after 300 simulation years in the hybridization experiment. Darker colours denote relatively higher densities, measured as number of individuals per patch. Results shown for select hybridization propensities: (a) 0%; (b) 1%; (c) 10%; (d) 100%

Considered spatially, *Z. silvanus* colonizes successively larger areas with increasing hybridization propensity. Without hybridization, its habitat remains restricted to the montane forest fragments, but it spreads through large parts of the landscape once hybridization occurs (Figure 3).

### 3.2 Habitat experiment

**FIGURE 4:**
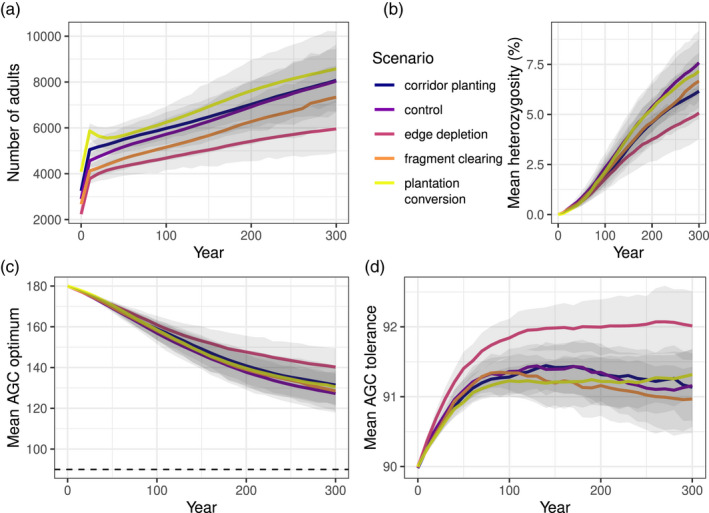
Development of key variables over 300 years in the habitat experiment, differentiated by habitat scenario. (a) Global number of adult *Z. silvanus* individuals. (b) Mean population heterozygosity of *Z. silvanus* (i.e. percentage of extraspecific chromosomes in the population gene pool). (c) Mean AGC optimum trait value of all *Z. silvanus* individuals. (d) Mean AGC tolerance trait value of all *Z. silvanus* individuals. Solid lines show the mean of 50 replicates, shaded areas are 95% confidence intervals. AGC: above‐ground carbon, in Mg C ha^−^ (a proxy for habitat type, see main text). The dashed line in panel (c) denotes the boundary between montane forest habitats (AGC ≧ 90) and other habitat types (AGC < 90)

In the habitat experiment, all scenarios showed a linear population growth of *Z. silvanus* after a 20 year burn‐in period. Final population sizes had a high variability, but depended on the amount of montane forest habitat in the scenario (Figure 4a, cf. Table S1). The rate of growth was almost constant between scenarios, with the exception of lower average growth in the edge depletion scenario.

Population heterozygosity also increased almost linearly, with the strongest increase in the control and the plantation scenarios, followed by the fragment clearing and corridors scenarios, and finally the edge depletion scenario. However, within‐scenario variance was very high compared with between‐scenario differences (Figure 4b).

Likewise, AGC optimum and AGC tolerance means showed strong overlaps between scenarios, with a steady drop in AGC optima and a slight increase in AGC tolerances. Again, the edge depletion scenario was the most distinct, showing both a stronger tolerance increase and a weaker optimum decrease (Figure 4c,d).

**FIGURE 5:**
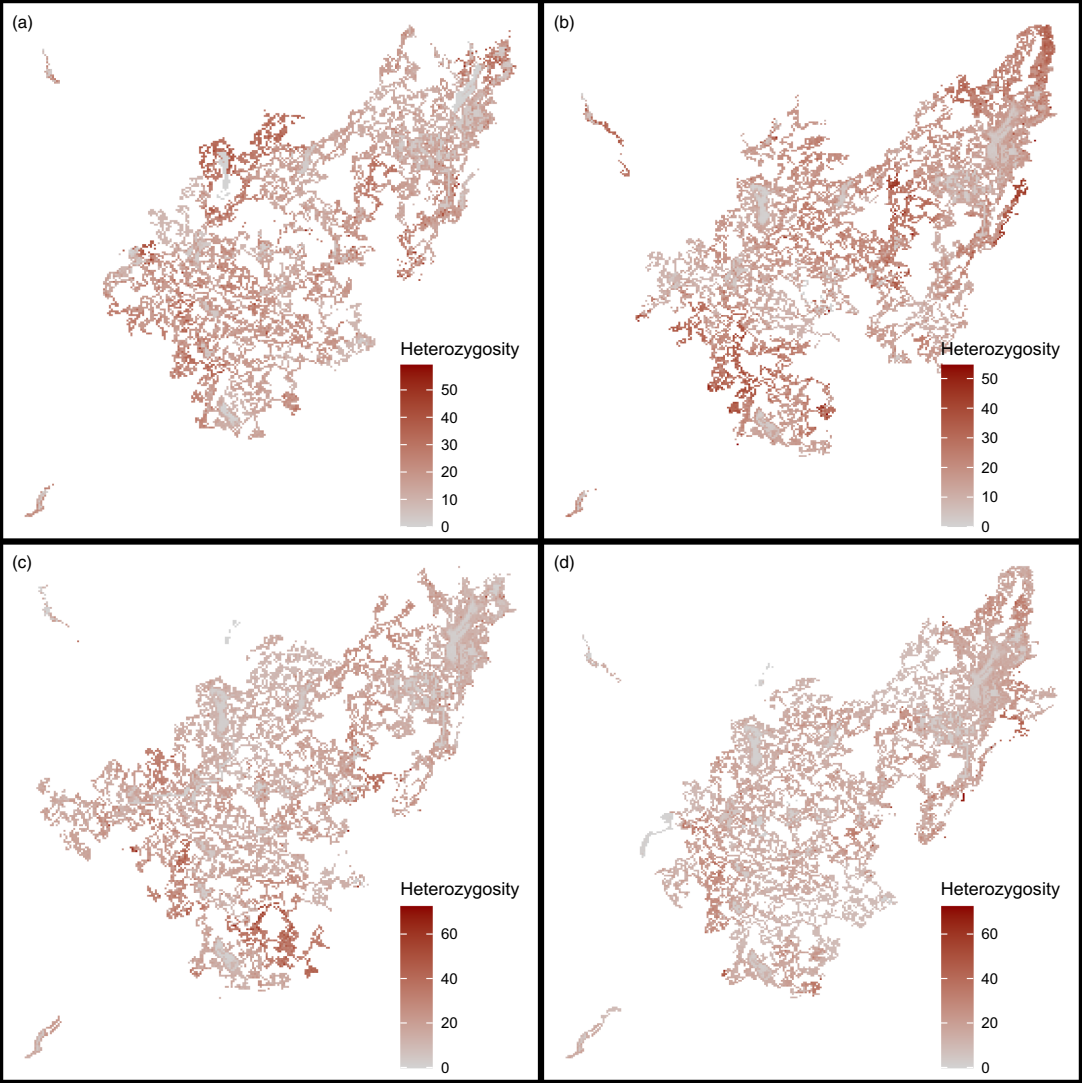
Spatial distribution of population heterozygosity of *Z. silvanus* in the Taita Hills, Kenya, after 300 simulation years in the habitat experiment. Darker colours denote relatively higher heterozygosity values, measured as percentage of extraspecific chromosomes in the patch gene pool. Grids show the four habitat change scenarios (excluding the control): (a) edge depletion; (b) fragment clearing; (c) corridor planting; (d) plantation conversion

The spatial analysis showed heterozygosity to be low in large montane forest fragments and higher in smaller fragments and the surrounding landscape (Figure 5). This was confirmed by the fragment‐level analysis (Table 1), which showed a negative correlation between fragment area and heterozygosity (linear regression of mean heterozygosity against log‐transformed area: slope − 1.8, adjusted *R* ^2^ = 0.31).

**TABLE 1:** Population heterozygosity in 20 montane forest fragments known to be inhabited by *Zosterops silvanus*

Fragment	Area	Scenario						
		Control	Edge depletion	Fragment clearing	Corridor planting	Plantation conversion	Mean	SD
Mbololo	180 ha	6.07%	3.60%	5.95%	4.81%	6.04%	5.29%	1.08
Ngangao	145 ha	4.98%	12.17%	3.97%	5.31%	4.24%	6.13%	3.42
Chawia	91 ha	6.64%	6.85%	6.48%	5.00%	6.69%	6.33%	0.76
Msidunyi	26 ha	5.53%	8.73%	7.13%	12.25%	0.91%	6.91%	4.18
Vuria extra 1	22 ha	10.19%	10.76%	10.98%	15.09%	0.71%	9.55%	5.31
Ronge	15 ha	10.83%	10.47%	10.52%	10.59%	10.57%	10.60%	0.14
Susu	14 ha	11.36%	13.76%	34.15%	12.63%	11.31%	16.64%	9.84
Fururu	8 ha	6.50%	7.08%	13.64%	7.19%	6.74%	8.23%	3.04
Vuria	7 ha	11.02%	8.45%	12.96%	14.76%	NA	11.80%	2.7
Ndiwenyi	5 ha	19.45%	19.85%	21.97%	17.27%	13.71%	18.45%	3.13
Macha E	4 ha	11.94%	12.75%	15.91%	10.03%	15.60%	13.25%	2.5
Susu extra 1	4 ha	17.99%	15.52%	22.63%	16.18%	12.69%	17.00%	3.68
Yale S	4 ha	6.45%	6.37%	5.86%	5.92%	14.21%	7.76%	3.61
Yale extra N	3 ha	16.05%	3.06%	5.86%	10.47%	10.24%	9.14%	4.96
Mwachora	2 ha	4.20%	1.94%	1.81%	1.28%	3.05%	2.46%	1.17
Kichuchenyi	1 ha	18.18%	28.69%	9.09%	16.48%	17.23%	17.93%	7.01
Wundanyi	1 ha	21.59%	14.77%	13.64%	11.24%	18.94%	16.04%	4.17
Yale extra 1	1 ha	18.10%	14.04%	14.65%	12.04%	18.54%	15.47%	2.78
Yale extra 2	1 ha	20.08%	15.91%	12.50%	23.30%	17.56%	17.87%	4.1

*Note:* Values give mean of 50 replicates for the listed habitat scenarios after 300 years. NAs indicate fragments that were not inhabited in the simulations.

Abbreviation: SD, standard deviation.

## 4 DISCUSSION

### 4.1 Introgressive hybridization and evolutionary rescue

As to our first question, the simulation results strongly indicate that evolutionary rescue is possible even at low levels of hybridization, as populations show strongly increasing trends as a consequence of introgressed genetic variation. Interestingly, population heterozygosity and population growth are not correlated linearly, so that intermediate heterozygosity levels already lead to high population growth (Figure 2a,b). This would mean that even a small amount of gene introgression can provide the genetic variability needed to adapt to novel environments.

In fact, the exploratory results suggest that in the longer term, populations with high hybridization propensities may not be as successful as those with a low‐to‐intermediate propensity (Figures S3 and S4). This agrees with our expectations and previous knowledge of hybridizing species complexes (Baskett & Gomulkiewicz, 2011). In our study, the cause for this is likely the adaptation trade‐off between AGC optimum and AGC tolerance. In the given model design and landscape, individuals with a low AGC optimum and a high AGC tolerance have the widest range of habitats available to them, giving them an evolutionary advantage. However, at the start of the simulation, *Z. flavilateralis* has a low AGC optimum but also a low AGC tolerance, while *Z. silvanus* has a high ACG tolerance but a high AGC optimum. Therefore, intermediate hybridization rates allow *Z. silvanus* to profit from the low AGC optimum of *Z. flavilateralis*, while not being swamped with the latter's low AGC tolerance (Figure 2c,d). Although this particular combination of traits is specific to the model, it illustrates well the general principle that hybridization may introgress both advantageous and disadvantageous alleles, and that whether the final result is favourable or not depends on the circumstances (Uecker et al., 2015).

It is notable that we clearly see hybrid vigour as an emergent property of our model. Although conservationists often worry about hybrid inferiority, which may lead to outbreeding depressions through maladaptive ‘intermediate’ phenotypes, the reverse phenomenon (hybrid vigour or heterosis) is actually more common (Bar‐Zvi et al., 2017; Rhymer & Simberloff, 1996; Rosas et al., 2010). In this study, hybrids develop a wider range of habitats and therefore a higher fitness than their parent species, which further encourages evolutionary rescue.

All the same, caution must be exercised when estimating the real‐world likelihood of evolutionary rescue by introgressive hybridization, as the results of exploratory analyses do show a strong effect of genetic linkage (Figure S5). Higher degrees of genetic linkage inhibit the selective introgression of individual traits discussed above, so that both beneficial and maladaptive alleles are taken up into the gene pool. This too ties in with previous results (Leidinger et al., 2021; Uecker et al., 2015). However, exploratory simulations also show that mutation‐based natural selection can also lead to evolutionary rescue, albeit slower than introgressive hybridization (Figure S6). Thus, at sufficiently high mutation rates, the cumulative effects of beneficial mutations may serve to balance out the detrimental effects of linkage.

Lastly, it should be pointed out that there is increasing empirical evidence that hybridization between *Z. silvanus* and *Z. flavilateralis* does indeed occur. In addition to the above‐mentioned indicators (mixed feeding flocks, high genetic diversity in *Z. silvanus*, general tendency of *Zosterops* to hybridize), new genomic analyses show a recent increase in the effective population size of *Z. silvanus*, which may be indicative of hybridization with *Z. flavilateralis* (see the Supplementary Material for details; cf. Engler et al., 2020). Hence, our findings will serve as a hypothesis for follow‐up genomic analyses once both genomes are adequately described.

### 4.2 Habitat structure, hybridization, and population growth

Contrary to our expectations for the second question, there was little influence of habitat configuration on population growth rates or global hybridization rates (Figure 4b). In addition, differences between final population sizes in the various habitat scenarios appeared to be purely due to area effects, with no noticeable impact of connectivity (Figure 4a).

The general importance of connectivity and dispersal are well‐known in conservation planning (e.g. Lens et al., 2002; Margules & Pressey, 2000). However, the movement behaviour of dispersing individuals and their response to the landscape is strongly species‐specific (Pe'er et al., 2011). One key difference between species is the extent to which they are willing to cross nonpreferred habitat (i.e. the matrix). The stochastic movement simulator, which we used as the basis for our dispersal submodel, does not have a penalty for cross‐matrix dispersal (Aben et al., 2014; Palmer et al., 2011). Hence, individuals will move through their preferred habitat if it is available, but will not avoid crossing large distances of matrix if it is not. This is an unrealistic assumption for many forest bird species in the Taita Hills, which will actively avoid open habitats (Aben et al., 2012). Habitat‐avoidance behaviour has the potential to strongly impact dispersal ability, and implementing it would therefore make connectivity much more important. The degree of habitat‐avoidance ought therefore to be considered as a parameter in all future studies using the stochastic movement simulator. In the case of *Z. silvanus*, however, there is good evidence that matrix dispersal does take place (Bytebier, 2001), and it has been measured to have the second‐highest dispersal rate of seven forest bird species studied in the Taita Hills (Lens et al., 2002). Accordingly, our finding of the low importance of connectivity in this context is not as improbable as it sounds at first, particularly as the distances between habitat fragments in the Taita Hills are within the dispersal range of *Z. silvanus*.

The distinctly worse performance of the edge depletion scenario (with lower population growth and heterozygosity, and higher AGC optimum; Figure 4) was probably due to the hard habitat boundary introduced by the scenario map. While the current montane forest fragments are often surrounded by medium‐AGC habitat, the modelled scenario replaced large parts of this with low‐AGC habitat (Figure 5a). As this habitat is outside the AGC range of *Z. silvanus*, this would have reduced the area in which hybridization can take place. Lower hybridization rates would then have led to worse habitat adaptation and lower population growth. In effect, this scenario proved to be a case of anthropogenic disturbance inhibiting gene flow for the species concerned (Crispo et al., 2011).

Apart from this edge depletion scenario, the other habitat scenarios appeared to have no significant effect on the hybridization rate. Indeed, population trajectories of habitat scenarios with different hybridization propensities mostly followed those seen in the hybridization experiment (compare Figures 2 and 4, Figures S7 and S8). Therefore, in this context, mating behaviour seems to be the dominant determinant of hybridization rates, rather than habitat configuration. The probable cause for this is that the main mechanism by which habitat change leads to increased hybridization is by bringing previously disjunct species closer together (Grabenstein & Taylor, 2018); whereas in this case, the two *Zosterops* species were already adjacent over a wide area. Moreover, as discussed above, the large dispersal distances and cross‐matrix dispersal of *Z. silvanus* made habitat connectivity much less important.

Although we did not find landscape‐level effects of habitat structure on hybridization in our tested scenarios, we did observe edge effects at the local level. We could show that population heterozygosity is lower in the interior of large fragments (Figure 5) and higher in smaller habitat fragments, which have a higher edge‐to‐area ratio (Table 1). Additionally, heterozygosity may be further reduced in large habitat fragments by the stabilizing selection caused by a larger standing variation in the local population's gene pool (Lopez et al., 2009). This suggests that the effect of habitat fragmentation on hybridization is scale‐dependent.

Future studies may extend our work by investigating the spatiotemporal interactions between anthropogenic land‐use change and bird population dynamics. We limited the scope of this study to static scenario maps as subsistence farming‐driven deforestation is a complex socio‐ecological process in its own right (Munthali & Murayama, 2012). However, coupling our ecological model with a land‐use change model would be a promising avenue to deepening our understanding of possible conservation trajectories in the Taita Hills (cf. Cabral et al., 2022).

## 5 CONCLUSION

Here, we show how an empirically parameterized individual‐based model can be used to provide qualitative predictions relevant to the conservation of an endangered species. Specifically, we explore the potential for evolutionary rescue within the context of a changing landscape, and present findings that may be validated by future genomic analyses and field monitoring studies.

Our results suggest that an endangered species in widespread contact with a congeneric in a fragmented landscape has a realistic chance of evolutionary rescue by introgressive hybridization. This is particularly true if hybrids show increased fitness (hybrid vigour), which happened in this case due to the increased generalism of hybrids relative to the parent species. In the long term, intermediate hybridization rates seem to be the most advantageous for population growth in these circumstances, due to trade‐offs between beneficial and deleterious introgressed alleles.

Our spatial analyses show that when gauging the effects of habitat change on populations of endangered species, it is crucial to consider the dispersal behaviour of said species at an individual level. In the case of highly dispersive species in a compact but fragmented landscape, it will be more important to increase overall habitat area rather than connectivity. Additionally, translocations of closely related but better adapted individuals into an at‐risk population (i.e. targeted gene flow) could be a promising approach to encourage evolutionary rescue.

In summary, this study shows that evolutionary rescue by introgressive hybridization is possible and, for *Z. silvanus*, even likely. The type of assessment made in this study highlights the importance of combining both genetic and ecological data to bring the predictive power of individual‐based ecological models to bear on open questions and current challenges in conservation.

## CONFLICT OF INTEREST

The authors declare no conflict of interests.

## Supporting information


Appendix S1

## Data Availability

The GeMM source code and relevant input files are archived on Zenodo (Leidinger & Vedder, 2021). The development version is available at https://github.com/CCTB‐Ecomods/gemm.
