# Supplementary material for: Hybridization may aid evolutionary rescue of an endangered East African passerine
Source: Evol Appl. 2022 Jul 4;15(7):1177–88. doi: 10.1111/eva.13440 (PMC9309464; doi:10.1111/eva.13440)
Supplement: Supplementary file 1 — Appendix S1 [file EVA-15-1177-s001.pdf]

Supplementary Material

"Hybridisation May Aid Evolutionary Rescue of an East African Passerine"

1 Model description

The following model description follows the ODD (Overview, Design concepts, Details) protocol (Grimm et al., 2010).

1.1 Purpose

The purpose of this model is to study the population development and genetics of *Z. silvanus* under different assumptions of mating behaviour and habitat change.

1.2 Entities, state variables, and scales

The primary entities of the model are *patches* and *individuals* (Listing 1).

Patches represent discrete one-hectare areas of ground and are characterised by an ambient temperature (in Kelvin), a measure of habitat type (above-ground carbon, in MgC ha^{-1}), and a carrying capacity (maximum number of adult individuals). They contain a community of individuals, i.e. the birds that breed in that area and their juvenile offspring.

Individuals refer to individual birds belonging to either of the species *Z. silvanus* and *Z. flavilateralis*. Each individual has a genome consisting of multiple genes, which each code for a specific trait. Genes are grouped into linkage units (chromosomes), although for the purposes of most experiments in this study we configured the model to create a separate linkage unit for each gene. Individuals are diploid, so their phenotype for each trait is determined by the mean value of the two genes coding for this trait. These traits determine an individuals adaptation to its habitat (i.e. temperature and above-ground carbon) and influence life-history processes like dispersal and reproduction (Fig. S1). Individuals are assigned a sex at birth and may find a partner after dispersal.

The model simulates the landscape of the Taita Hills (total area: $962\,km^2$, or 96,170 patches) over 300 years, with one time step per year.

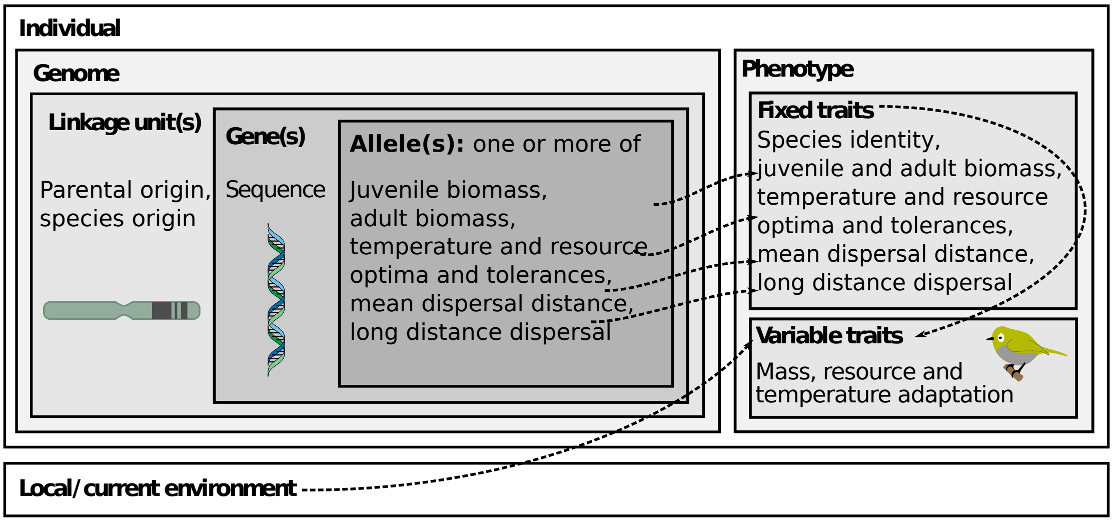

Figure S1: Relationship of genotype and phenotype in GeMM individuals. Note that the model was used in a simplified setup in this study, so not all of the mentioned traits are relevant—see text for details. (Modified from Leidinger et al., 2021.)

1.3 Process overview and scheduling

Patches and their communities are created during initialisation. The patch environment stays constant, but each year, each patch community undergoes the following processes (in order):

1. **Establishment:** Individuals that dispersed in the previous year are checked for genetic viability. Any individuals that are not viable due to extreme low fitness or genetic defects are killed.
2. **Survival:** Density-independent mortality of individuals.
3. **Reproduction:** Each breeding pair in a patch produces up to two offspring. Parent genomes undergo meiosis, leading to recombination in the offspring genomes.
4. **Mutation:** All juveniles in a patch have random changes introduced to their gene sequences and trait values. (Turned off by default.)
5. **Dispersal:** Juveniles leave the parent patch, iteratively moving to the neighbouring patch whose habitat is closest to their optimum. If they find a patch with an available mate or a free territory, they join the patch community. If they reach their maximum dispersal distance without settling down, they die.

1.4 Design concepts

Basic principles. To estimate unknown parameters and process rates, GeMM uses the Metabolic Theory of Ecology (MTE; Brown et al., 2004). This describes biological rates such as reproduction, mortality, and mutation as a mathematical function of temperature

and individual body mass. As we have good data for most *Zosterops* life processes, and to avoid additional complexity, we only applied the MTE to the mutation submodel in this study.

Emergence. The spatial and temporal demographics and population genetics of the two *Zosterops* species are emergent properties of the model, arising from the interaction of individuals with each other and the landscape.

Objectives. During dispersal, individuals look for patches with suitable habitats based on their above-ground carbon (AGC) preference. Goodness of fit is defined as the difference between patch AGC and individual optimum AGC, with smaller values being better.

Sensing. During dispersal, individuals perceive the AGC values of each neighbouring patch (i.e. 100 m sight radius). Within the current patch, individuals sense the availability of free space and the suitability of another individual as a partner.

Interactions. A male and a female individual can form a breeding pair, exclusively mating with each other while both are alive. Indirect interaction takes place in the form of competition for space and partners, as individuals that fail to find an available territory or partner die.

Stochasticity. Random components influence the initial population sizes, the likelihood of per-individual density-independent mortality, the reduction of the diploid genomes into haploid gametes during meiosis, and the number of offspring per breeding pair per year.

Observation. The model records per-patch population sizes over time, as well as the distribution of trait values and the degree of heterozygosity in each patch population.

1.5 Initialisation

The first step of model initialisation is to read in all model parameters (see below). These include species definitions for all *Zosterops* species that are to appear in the model, which are used to create species archetype objects.

Following this comes the process of reading in the user-defined map file. This specifies the properties of each individual patch, including which patches are to be initialised with a community, and the local carrying capacity. Once the patch object has been created, if it is to have an initial community, the model checks whether its habitat is suitable for any of the predefined *Zosterops* species.

If that is the case, the initial community size for the patch is determined at random. The community is then created by copying the suitable archetypes, always generating an

equal number of males and females to form the first breeding pairs. After copying, the new individuals are mutated to give rise to the initial population variability.

1.6 Input data

GeMM takes two external input files for each run: the configuration file with relevant model parameters, and the map file specifying the simulation world.

The configuration file can include all parameters listed in Appendix 1.8. Parameters that are not explicitly configured revert to the default, as defined in the source code. Notable parameters include the fertility rate, mean dispersal distances, and hybridisation propensity, as well as the species definitions mentioned above. Also, a seed for the random number generator can be set, allowing exact replicates to be run. (Simulations with identical seed and input and will be identical in output.)

The default map file for the Taita Hills (cf. Fig. S2) was generated from remote sensing AGC data as published in Adhikari et al. (2017) and Pellikka et al. (2018). The original data was obtained in 2015 through a combination of airborne laser scanning, satellite imagery, and field work at a resolution of $32 \times 32\,\mathrm{m}$. As we assumed one-hectare patches for GeMM, we upscaled this to $100 \times 100\,\mathrm{m}$ using cubic convolution resampling in QGis (QGIS.org, 2020).

The carrying capacity K (in birds/ha) for each patch was calculated from the patch's above-ground carbon value P_{AGC} as:

$$K(P) = \lfloor \frac{P_{AGC} + 10}{0.05 P_{AGC} + 20} \rfloor \times 2 \tag{1}$$

This equation was chosen to relate the AGC ranges of different habitat types (Pellikka et al., 2018) to observed breeding densities of *Zosterops* in these habitats (J. Engler, personal communication; Mulwa et al., 2007). It gives a density of one breeding pair per hectare in woodland, 2–3 pairs/ha in exotic forest, and 4–8 pairs/ha in montane forest. To account for very low population densities in the savannah (observed as one breeding pair per square kilometer), patches with $P_{AGC} < 10$ had a 1 % chance of being assigned a carrying capacity of 2.

The scenario maps for the habitat experiments were based on the same data as the default map, but edited to reflect the scenarios described in Section 2.2. (Table S1). Note that the landscape was not dynamic, i.e. scenario maps stayed constant and there was no habitat change throughout the simulation. (While GeMM is capable of using multiple maps over a single simulation run, but we decided not to use this feature due to the complexities of modelling land-use change—see main text.)

1.7 Submodels

All relevant model parameters are listed in Table S2, and will be referred to in the following using `block print`. For a comprehensive list of parameters (including those not relevant to the study presented here), see Listing 2.

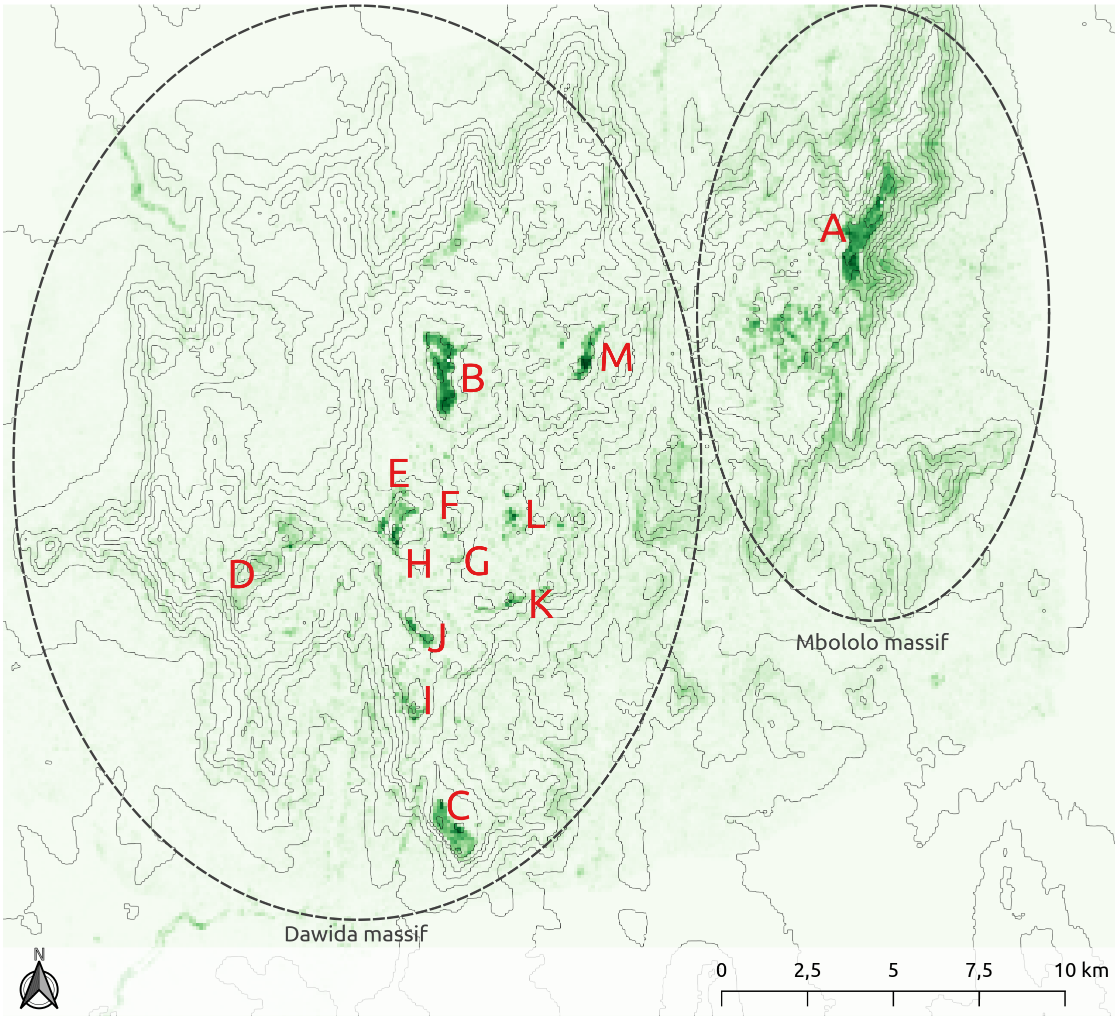

Figure S2: Map of the study region in the Taita Hills. Contour lines have a vertical interval of 100 m. Darker green means higher above-ground carbon, the darkest areas are montane forest fragments. Letters designate exotic or montane forest fragments: A Mbololo, B Ngangao, C Chawia, D Vuria, E Yale, F Wesu, G Mbili, H Weni Mwana, I Susu. The names of the fragments J–M are not known. (AGC data from Pellikka et al., 2018; elevation data from "Hole-Filled Seamless SRTM Data V4", 2018).

Table S1: Details of map changes in the habitat scenarios. For location of forest fragments, see Fig. S2. For the control scenario, measures of montane forest area and global carrying capacity (combined for both *Zosterops* species) give the map totals. For the other scenarios, the difference to the control scenario is given.

	Scenario	Reference	Fragments involved	Montane forest area	Global carrying capacity
**1**	*Control*	from Pellikka et al. (2018)	—	681 ha	110,796 individuals
**2**	*Edge depletion*	cf. Teucher et al. (2020)	Mbololo, Ngangao, Chawia	–149 ha	–1582 individuals
**3**	*Fragment clearing*	cf. Pellikka et al. (2013)	Susu, J, K, L	–58 ha	–634 individuals
**4**	*Corridor planting*	from Wagura (2018)	Vuria, Yale, Ngangao	+85 ha	+552 individuals
**5**	*Plantation conversion*	from Githiru et al. (2011)	Susu, Vuria, Wesu, Weni Mwana, Mbili	+294 ha	+1136 individuals

Establishment. Establishment is carried out for all individuals that are new to a patch following dispersal by testing their genetic viability. This includes ensuring that its genome codes for all necessary traits, and that all trait and adaptation values are within acceptable bounds (e.g. >0). If the individual fails any of these tests, it is removed from the patch community and dies.

Survival. Survival represents density-independent mortality of all adult individuals (i.e. post-dispersal, post-establishment). Each year, every adult has a death probability determined by the global `mortality` parameter. The value for this was chosen to reflect known life expectancies of related *Zosterops* species (Bird et al., 2020).

Reproduction. Every adult, established individual with a partner reproduces every year. The number of offspring per breeding pair per year is a randomly chosen integer between 0 and `fertility`. This value takes into account observed *Zosterops* clutch sizes and annual breeding attempts as well as nestling and fledgling mortality rates. It thus represents the number of juveniles who survive until post-natal dispersal.

For each offspring individual, the parent genomes undergo meiosis, producing two randomly assembled haploid chromosome sets that are combined to form the offspring genome. Offspring are assigned a random sex and the species of their parents. In the case of hybrid offspring (with parents of different species), the species of the parent they are phenotypically closer to is chosen.

Mutation. If the `mutate` parameter is true, each juvenile's genome is mutated after birth. Mutations randomly change individuals' phenotypes and may thus be detrimental, neutral, or beneficial, depending on the environment. The number of mutations per individual $U(I)$ is calculated using the Poisson distribution as:

$$U_P(I) = \text{Poisson}\Big(\mu \times e^{-E_{act}/(k_B \times P_{temp})}\Big) \tag{2}$$

where μ is the global parameter `mutationrate`, which is followed by the metabolic coefficient with the activation energy E_{act}, the Boltzman constant k_B, and the patch temperature P_{temp} (cf. Appendix 1.8; Brown et al., 2004).

Each mutation is then carried out by choosing a random gene and changing one of its nucleotides. Simultaneously, the trait that it codes for is changed using the normal distribution:

$$T_m = \text{Normal}\Big(T_o, T_o \times \varphi\Big) \tag{3}$$

where T_m is the mutated trait value, T_o is the original trait value, and φ is the global parameter `phylconstr`.

Note that mutations were turned off for this study, except during one exploratory scenario (see Section 2.1).

Dispersal. The model simulates post-natal dispersal of all juveniles in the year they are born. Secondary (i.e. adult) dispersal is not simulated.

The dispersal algorithm is a simplified version of the Stochastic Movement Simulator (SMS) developed by Palmer et al. (2011). This algorithm has been found to produce realistic dispersal patterns when tested against real movement data of two other forest bird species in the Taita Hills (Cabanis' greenbul *Phyllastrephus cabanisi* and white-starred robin *Pogonocichla stellata*; Aben et al., 2014).

The maximum dispersal distance of each individual is drawn from a logistic distribution:

$$S(I) = \text{Logistic}\Big(I_{dispmean}, I_{dispshape}\Big) \tag{4}$$

where $I_{dispmean}$ is the individuals mean dispersal distance and $I_{dispshape}$ is the scale parameter. (For the archetype individuals, these values are initially set to `dispmean` and `dispshape`, respectively.)

The individual keeps moving until it either finds a suitable patch to settle in, or the maximum dispersal distance is reached, in which case it dies. During each movement step, it evaluates the difference between neighbouring patches' AGC value and its own AGC optimum, moving to the patch with the least difference. There is no penalty for moving to patches outside the individual's AGC tolerance range. Patches that have already been visited are ignored.

If the current patch is within its AGC tolerance range, the individual checks for an empty territory (i.e. two empty slots in the patch capacity) or an available mate. An individual already living in that patch is an available mate if it is of the opposite sex, does not have a partner, and there is a free slot in the patch capacity. Individuals first search for available mates among conspecific individuals. If there are no conspecific available mates and the `speciation` parameter is set to *off*, the individual then searches for a non-conspecific mate, accepting a potential mate with a probability determined by the `tolerance` parameter (i.e. the hybridisation propensity).

If a suitable patch is found, the individual is added to the patch community. If there is an available mate, the two individuals are assigned each other as partner, thus forming a new breeding pair. Breeding pairs stay faithful for life, but individuals whose partners die can form new breeding pairs with younger individuals that disperse into their patch.

1.8 Implementation details

This appendix provides further details on model parameters and variables.

The full model source code along with the scripts that were used to set up and analyse the experiment may be found on the accompanying CD ROM, or online on Github at `https://github.com/CCTB-Ecomods/gemm`. The raw data has been archived on the CCTB servers under `/storage/ecomod/zosterops/hybridstudy`.

Listing 1 shows the full list of state variables for individuals and patches. Listing 2 contains all model parameters and their generic value (for values used in the current study, see Table S2).

GeMM uses the following physiological constants:

Boltzmann constant: $1.38064852 \times 10^{-23} JK^{-1}$ (k_B)

Activation energy: $10^{-19} J^{-1}$ (E_{act}; Brown et al., 2004)

Table S2: List of model parameters and default values. The values of `maps` and `tolerance` (i.e. hybridisation propensity) were varied in the main experiment scenarios, while each replicate was initialised with an individual `seed`. Parameters that are not biologically relevant to the Zosterops study are excluded; for more details see Listing 2. *(continued on next page)*

**Parameter**	**Default value**	**Explanation**	**Justification / Reference**
`seed`	0	Initialisation value for the random number generator.	cf. Saito and Matsumoto (2008)
`maps`	taita_hills.map	Names of map file containing the patch definitions.	Section 1.6
`outfreq`	10	Frequency of output generation (data is saved every `outfreq` years).	tradeoff between analytical precision and computational cost
`linkage`	none	Degree to which genes are joined into chromosomes. Options: "none" (one gene per chromosome), "full" (one chromosome per haploid genome), "random" (random number of genes per chromosome).	"none" allows for maximal genetic admixture
`nniches`	2	Number of environmental niches modelled.	First niche is temperature, second niche above-ground carbon.
`mutate`	false	Turn on the mutation process?	Section 1.7
`phylconstr`	0.1	Standard deviation of trait value shift during mutation	arbitrary (not needed unless `mutate` is true)
`mutationrate`	3.6×10^{10}	Scaling parameter of mutation probability, see Section 1.7	gives 0–7 mutations per individual
`indsize`	adult	At initialisation, create individuals as "adults", "juveniles", or "mixed"?	avoid long burn-in period
`degpleiotropy`	0	Degree of pleiotropy and multigenic inheritance (0 = none)	reduce implementation complexity
`speciation`	off	Allow species to split up into mutually incompatible lineages?	reduce analytical complexity
`mode`	"zosterops"	Switch between GeMM modes ("default", "invasion", or "zosterops")	

Table S2: *(continued from previous page)*

Parameter	Default value	Explanation	Justification / Reference
`tolerance`	0.1	Likelihood of accepting a non-specific mate if no conspecific mate is available	Section 1.7
`capgrowth`	true	prevent individuals from becoming larger than their reproductive size	biological realism for birds
`cellsize`	8	Global default patch carrying capacity (birds/patch)	Mulwa et al. (2007)
`fertility`	2	Maximum number of offspring per breeding pair per year	J. Engler (personal communication), Jetz et al. (2008)
`dispmean`	18	Initial mean dispersal distance in patch lengths	L. Lens (personal communication)
`dispshape`	2	Initial dispersal kernel shape parameter	gives effective dispersal distances of 0.5–3 km
`maxrepsize`	12	Maximum reproductive size of initialised species (g)	L. Lens (personal communication)
`minrepsize`	10	Minimum reproductive size of initialised species (g)	L. Lens (personal communication)
`maxseedsize`	7.4	Maximum birth size of initialised species (g)	physiologically irrelevant, but must be smaller than the reproductive size
`minseedsize`	4.5	Minimum birth size of initialised species (g)	
`metabolicdeath`	false	Use the MTE to calculate mortality rates?	
`mortality`	0.125	Global annual mortality rate of adult individuals	Bird et al. (2020)
`heterozygosity`	true	measure and record population heterozygosity	
`species`	[(lineage:"silvanus", precopt:180, prectol:90, tempopt:293, temptol:2), (lineage:"flavilateralis", precopt:50, prectol:47, tempopt:293, temptol:2)]	*lineage*: species name<br>*precopt*: AGC optimum<br>*prectol*: AGC tolerance<br>*tempopt*: temperature optimum<br>*temptol*: temperature tolerance	AGC ranges from Pellikka et al. (2018), temperature ranges chosen to fit map

Listing 1: Source code excerpt showing state variables of the primary entities *Patch* and *Individual*. Note that in former model versions, precipitation was used instead of above-ground carbon (related variables were not renamed).

```julia
"""
One of the core structs of the model, representing an individual organism.
"""
mutable struct Individual
    lineage::String
    genome::Array{Chromosome, 1}
    traits::Dict{String, Float64}
    marked::Bool  # is individual new to a patch? (after dispersal or birth)
    precadaptation::Float64  # adaption to above−ground carbon
    tempadaptation::Float64  # adaption to temperature
    size::Float64 # body mass
    sex::Sex
    partner::Int  # ID of the partner individual (default 0)
    id::Int
end

"""
One of the core structs of the model, representing a one−hectare
patch of ground.
"""
mutable struct Patch
    id::Int
    location::Tuple{Int, Int}
    capacity::Float64   # carrying capacity in (individuals per patch)
    temp::Float64       # temperature (Kelvin)
    prec::Float64       # above−ground carbon (MgC/ha)
    nicheb::Float64     # additional generic niche − currently not used
    neighbours::Array{Int,1}        # indices of neighbouring patches
    community::Array{Individual, 1} # collection of adult individuals
    seedbank::Array{Individual, 1}  # collection of juvenile individuals
    initpop::Bool       # initialise with a population?
    isisland::Bool      # island? (if false −> mainland)
    invasible::Bool     # can exotics land here?
    isolated::Bool      # add a distance penalty when dispersing?
end
```

Listing 2: Complete list of all GeMM model parameters and default values as defined in the source code. Note that many of these are not relevant (or different) for the Zosterops study. Compare table S2. *(continued on next page)*

```
"""
    defaultSettings()

Defines the list of configuration variables and returns their default values
in a Dict.
"""
function defaultSettings()
    # Return the default settings. All parameters must be registered here.
    Dict(
        "biggenelength" => 200, # length of the compatibility gene's sequence (
            if `usebiggenes`)
        "borders" => "absorbing", # border behaviour: absorbing/reflecting/
            mainland
        "burn-in" => 1000, # timesteps before invasion starts
        "capgrowth" => false, # strictly limit individuals' size to `repsize`
        "cellsize" => 20e6, # maximum biomass in g/ha (cf. Clark et al. 2001)
        "compressgenes" => true, # save gene sequences as integers to reduce
            memory usage
        "config" => "", # configuration file name
        "debug" => false, # write out debug statements
        "degpleiotropy" => 0.1, # How frequent are pleitropy and polygenic
            inheritance? 0 <= degpleiotropy < 1
        "dest" => string(Dates.today()), # output folder name
        "dispmean" => 1.0, # maximum value of randomly drawn mean dispersal
            distance in cells
        "dispshape" => 1.0, # maximum value of randomly drawn shape parameter
            for dispersal kernel. determines tail fatness/long distance
            dispersal
        "disturbance" => 0, # percentage of individuals killed per update per
            cell
        "fasta" => "off", # record fasta data? "off", "compat", "all" (high
            detail output)
        "fertility" => exp(28.0), # global base reproduction rate (cf. Brown et
            al. 2004)
        "fixtol" => true, # fix mating tolerance globally to `tolerance`
        "global-species-pool" => 0, # size of the invasion species pool
        "globalmating" => false, # global pollen "dispersal"
        "growthrate" => exp(25.2), # global base growth (cf. Brown et al. 2004)
        "heterozygosity" => false, # keep track of heterozygosity when studying
            hybridisation
        "indsize" => "seed", # initialize organisms as seed, adult or mixed
        "isolationweight" => 3.0, # additional distance to be crossed when
            dispersing from or to isolated patches
        "lineages" => false, # record lineage and diversity data (low detail
            output)
        "linkage" => "random", # gene linkage type (random/full/none)
        "logging" => false, # write output to logfile
        "maps" => "", # comma-separated list of map files
        "maxbreadth" => 5.0, # maximum niche breadth
        "maxloci" => 1, # maximum number of loci/copies per gene
        "maxprec" => 10.0, # max optimum precipitation
```

```
        "maxrepsize" => 14.0, # maximal repsize in grams is exp(maxrepsize) ->
            1.2 t
        "maxseedsize" => 10.0, # maximal seedsize in grams is exp(maxseedsize)
            -> 22 kg
        "maxtemp" => 313.0, # max optimum temp in K
        "metabolicdeath" => true, # if false, `mortality` gives absolute per-
            update mortality probability
        "minprec" => 0.0, # min optimum precipitation
        "minrepsize" => 3.0, # minimal repsize in grams calculated as exp(
            minrepsize) -> 20 g
        "minseedsize" => -2.0, # minimal seedsize in grams calculated as exp(
            minseedsize) -> 0.14 g
        "mintemp" => 273.0, # min optimum temp in K
        "mode" => "default", # experiment type ("default", "invasion", or "
            zosterops")
        "mortality" => exp(22), # global base mortality (cf. Brown et al. 2004)
        "mutate" => true, # mutations occur
        "mutationrate" => 3.6e10, # one mutation per generation/individual,
            corrected for metabolic function
        "nniches" => 2, # number of environmental niches (max. 2)
        "outfreq" => 10, # output frequency
        "overfill" => 1.0, # modifier of cellsize to allow over-filling capacity
        "phylconstr" => 0.1, # phylogenetic constraint during mutation and inter
            -loci variation. scales trait value as sd.
        "popsize" => "metabolic", # initialisation algorithm: metabolic/bodysize
            /minimal/single
        "precrange" => 0, # max optimum precipitation - deprecated! (use `
            maxprec`)
        "propagule-pressure" => 0, # number of non-native individuals introduced
             per invasion event
        "quiet" => false, # don't write output to screen
        "raw" => true, # record raw data
        "sdprec" => 0.0, # SD of precipitation change per time step
        "sdtemp" => 0.0, # SD of temperature change per time step
        "seed" => 0, # for the RNG, seed = 0 -> random seed
        "smallgenelength" => 20, # standard gene sequence length (max. 21)
        "speciation" => "neutral", # allow lineage differentiation? off/neutral/
            ecological
        "species" => Dict{String,Any}[], # define trait values for each
            Zosterops species
        "static" => false, # whether mainland sites undergo eco-evolutionary
            processes (implies "mainland" borders)
        "stats" => true, # record population statistics (medium detail output)
        "tolerance" => 0.8, # sequence similarity threshold for reproduction if
            `fixtol` == true
        "traitnames" => ["compat", "dispmean",
                         "dispshape", "numpollen",
                         "precopt", "prectol",
                         "repsize", "seqsimilarity",
                         "selfing", "seedsize",
                         "tempopt", "temptol"], # minimal required traitnames
        "usebiggenes" => true, # use a longer sequence for the compatibility
            gene
    )
end
```

2 Exploratory scenarios

2.1 Experimental design

We conducted a number of exploratory experiments to investigate the effects of various model assumptions on the simulation results.

First, we wanted to explore how populations were likely to develop beyond the 300-year time frame chosen for the main experiments. Accordingly, we ran a repeat of the hybridisation experiment over 1000 years. As stated in the main text, we chose the main 300-year duration because we expected it to be long enough to allow eco-evolutionary dynamics, while not being too long for conservation concerns. We particularly note that ongoing land-use change is expected to make the habitat maps used in our simulations increasingly unrealistic the further they are projected into the future. However, there is theoretical interest in exploring longer-term population dynamics, even if the obtained results cannot be taken as meaningful for conservation planning. Specifically, this allows us to gauge how close populations are to reaching equilibrium by the end of the main study period.

Second, although this study deals with evolutionary rescue, we disabled mutations by default to prevent conflating effects. Nonetheless, mutations are of course important and ought to be considered. Therefore, to compare the effect size of introgressive hybridisation with that of mutation-based natural selection, we simulated three scenarios in which mutation was turned on. For these, we set the hybridisation propensity to 0, while varying the mutation rate parameter between 0, 3.6×10^9, 3.6×10^{10}, and 3.6×10^{11} (giving a maximum of 0, 3, 6, and 21 mutations per individual, respectively; see section 1.7). Mutations in GeMM may be detrimental, neutral, or beneficial depending on the individual's environment, and are therefore subject to natural selection.

Third, in the main study we also assumed a simplified genetic architecture with no linkage, i.e. one gene per chromosome (Section 1.2). Again, this was done to prevent conflating effects. However, it has been shown that genetic architecture can have a strong effect on a species' adaptive potential (Schiffers et al., 2014; Uecker et al., 2015); and a previous study with GeMM showed a clear selective benefit of low linkage, due to the increased recombination possibilities of unlinked genes (Leidinger et al., 2021). Hence, to verify that the assumption of no linkage did not qualitatively affect the results of the study, we ran additional scenarios with full linkage (where a single chromosome held all genes of a haploid genome) and random linkage (varying numbers of chromosomes). For these scenarios, mutations were turned off and the hybridisation propensity set to 0.01.

Finally, we twice repeated the habitat experiment as described in the main text, but set the hybridisation propensity to 10 % and 0 %, respectively, rather than 1 %.

Each of these exploratory experiments was run with 10 replicates.

2.2 Results

When simulating the hybridisation experiment over 1000 years, population levels plateaued after 300 years, with large overlaps between scenarios with hybridisation. Compared to the 300 year experiment, there were changes in the ranking of scenarios' population sizes, with intermediate hybridisation propensities doing slightly better than high hybridisation propensities. However, the differences remained small compared to the variability (Fig. S3a, S4). Population heterozygosity, AGC optimum, and AGC tolerance were largely the same as after 300 years, and kept the ranking by hybridisation propensity (Fig. S3b,c,d).

In the linkage experiment, scenarios with linkage (either full or random) were quite distinct from the no linkage scenario. Population growth occurred in all scenarios, but was strongest without linkage. Mean heterozygosity was higher with linkage, as was the AGC optimum. After a small initial rise, the AGC tolerance dropped again in scenarios with linkage, while maintaining its higher-than-initial level without linkage (Fig. S5).

In the mutation experiment, populations of *Z. silvanus* also grew over time, with more growth at higher mutation rates. Growth was slower than in the hybridisation experiment, but, at the highest mutation rate, reached much higher population levels (Fig. S6a). As in the hybridisation experiment, AGC optimum dropped steadily; however, AGC tolerance rose in all scenarios (Fig. S6c,d).

The repeat of the habitat experiment with a hybridisation propensity of 10 % yielded qualitatively the same results as with a hybridisation propensity of 1 %, and closely followed the trajectories of the hybridisation experiment at 10 % hybridisation propensity (Fig. S7). At 0 % hybridisation propensity, no intermixing of species took place, and population development consequently mirrored that seen in the hybridisation experiments in the equivalent scenario, although the final population size reached depended on the amount of montane forest habitat available (Fig. S8).

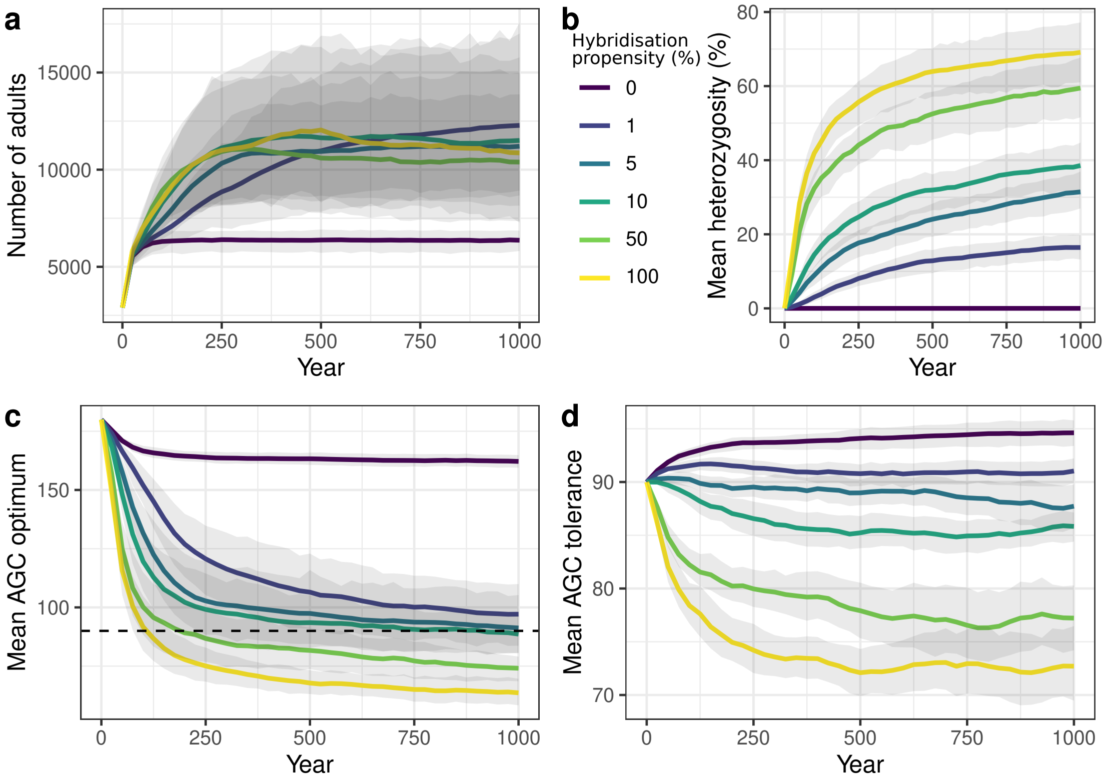

Figure S3: Population development of *Z. silvanus* under various hybridisation propensity settings over 1000 years. Solid lines show the mean of 10 replicates, shaded areas are 95 % confidence intervals.

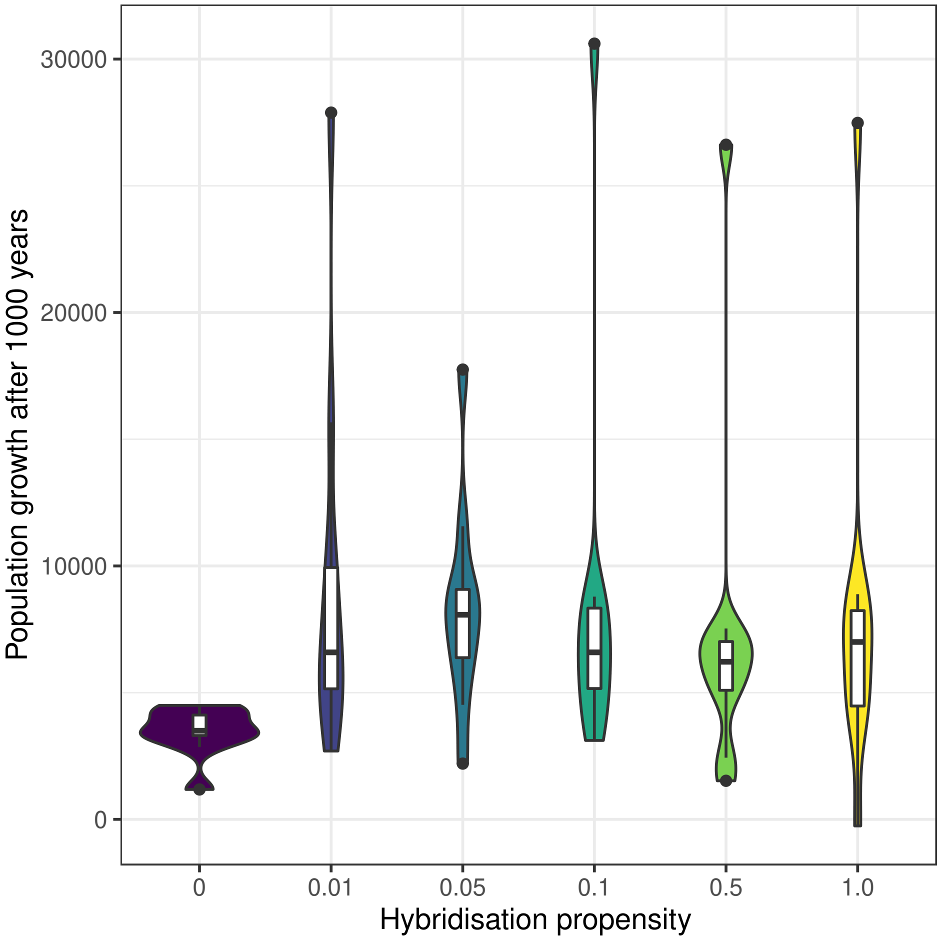

Figure S4: Population growth of *Z. silvanus* after 1000 years in the long hybridisation experiment. Violin plots show the distribution of values over 10 replicates, inset boxplots denote median and interquartile range.

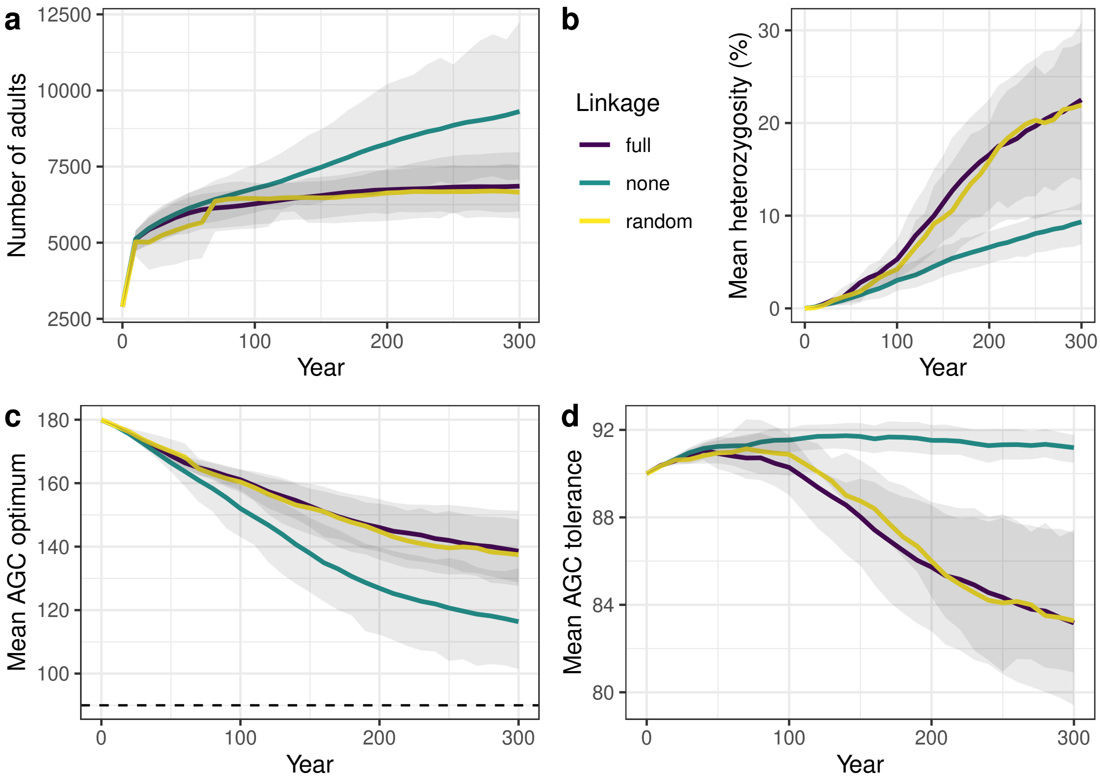

Figure S5: Population development of *Z. silvanus* under various genetic linkage settings, using a hybridisation propensity of 10 % and no mutations. Solid lines show the mean of 10 replicates, shaded areas are 95 % confidence intervals.

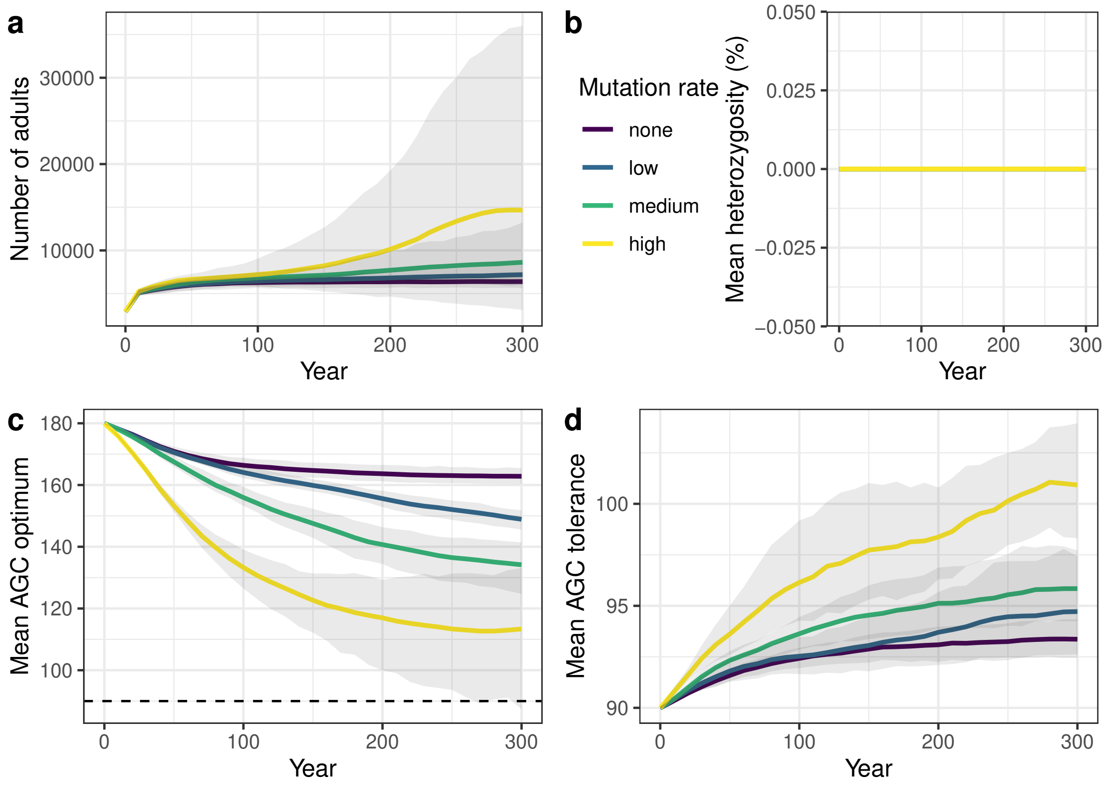

Figure S6: Population development of *Z. silvanus* under various mutation rate settings, using a hybridisation propensity of 0 % and no linkage. Solid lines show the mean of 10 replicates, shaded areas are 95 % confidence intervals.

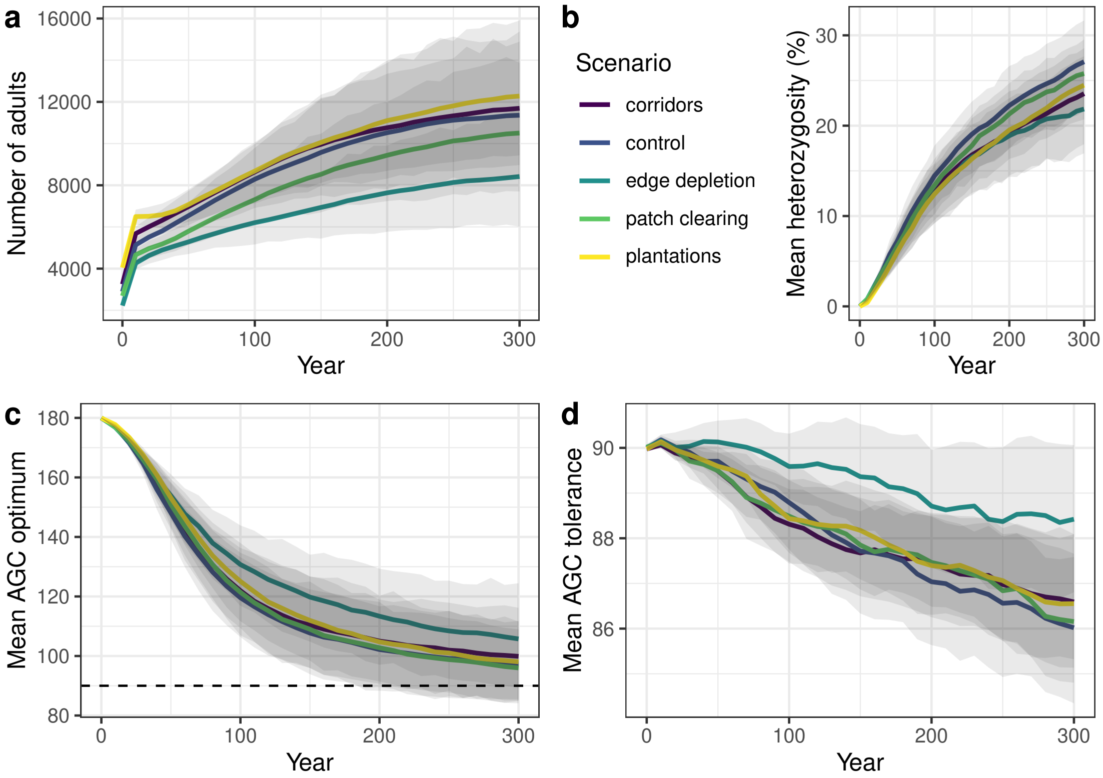

Figure S7: Population development of *Z. silvanus* in a rerun of the habitat experiment, using a hybridisation propensity of 10 %. Solid lines show the mean of 10 replicates, shaded areas are 95 % confidence intervals.

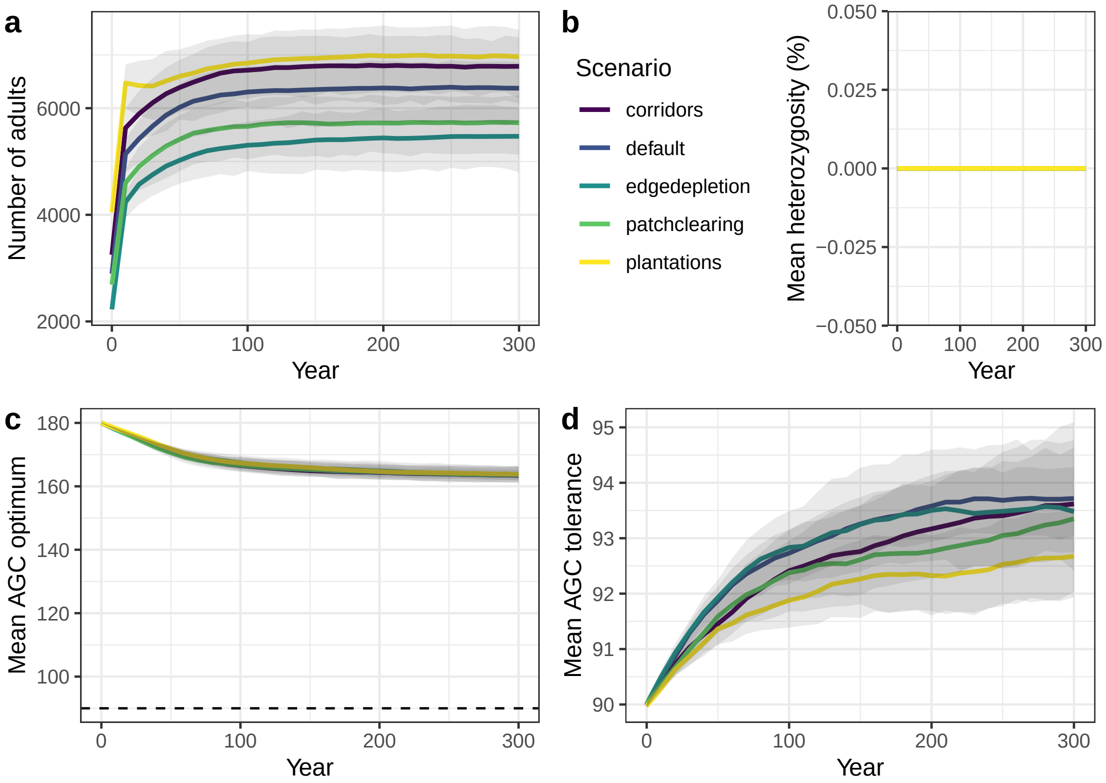

Figure S8: Population development of *Z. silvanus* in a rerun of the habitat experiment, using a hybridisation propensity of 0 %. Solid lines show the mean of 10 replicates, shaded areas are 95 % confidence intervals.

3 Genomic analysis

3.1 Methods

Genomic analysis of effective population size:

To allow comparisons from our simulation results to genomic inferences, we performed Pairwise sequential Markovian coalescent (PSMC) analyses (Patton et al., 2019) of historical effective population size (N_e) to test for signatures of potential hybridization between the highland *Z. silvanus* and its lowland congener *Z. flavilateralis*. We analysed five individuals of four closely related Zosterops species across Kenya, including three species inhabiting highland sky-island systems; *Z. kulalensis*, *Z. mbuluensis* and *Z. silvanus* (one individual from stable habitat in Mt. Kasigau and one from a remnant forest fragment surrounded by disturbance in the Taita Hills, Fig. S2 location J at ~1760m asl), and the *Z. silvanus* lowland congener, *Z. flavilateralis*.

Genomic DNA was extracted from blood samples using a salt extraction protocol and whole genomes sequenced using paired-end Illumina on the NovaSeq 6000, generating high genome read coverage (>18x per individual). Raw reads were trimmed using cutadapt (v 2.10) and aligned to a *Zosterops borbonicus* reference genome (Leroy et al., 2019), using the BWA mem algorithm with default settings (Li & Durbin, 2009). Duplicate reads were marked using Picard MarkDuplicates (v 2.23.3). Mapping was checked using Qualimap (v 2.2.1). Resulting bam alignment files were used to generate consensus genome sequences for each individual (fastq) using the `mpileup` command in SAMtools and the `vcf2fq` command from `vcfutils.pl`, with the *Zosterops borbonicus* genome assembly as the reference. Each fastq file was filtered for sequencing errors by excluding sites at which the per-site root-mean-square mapping quality was below 25, the inferred consensus quality was below 20, and the variant read depth was less than 10x or more than twice the average across the genome. PSMC analyses were performed using the following fixed parameters across each individual: maximum number of iterations (N) of 30, maximum coalescent time (t) of five, initial theta/rho ratio (r) of one and parameter pattern (p) of '`4+30*2+4+6+10`'. These values were chosen in line with PSMC analyses conducted across other avian species (36 avian species; see Nadachowska-Brzyska et al., 2015). To scale the PSMC output to real time we use estimates of generation time as one year (as in main text) and neutral mutation rate as 0.2×10^{-8} (Smeds et al., 2016) in the `psmc_plot.pl` command.

3.2 Results

Genomic inference of highland-lowland hybridization in isolated patches:

The PSMC results provide some genomic evidence of demographic histories in highland-lowland White-eyes (*Zosterops sp.*). The lowland congener *Z. flavilateralis* shows highest effective population size (N_e) with a peak at around 100 kya before decreasing. Independent of divergence times, sky-island specialists sharply lose N_e and converge at comparably low levels (i.e. $N_e < 10^5$). Interestingly, there is a separation of the otherwise highly

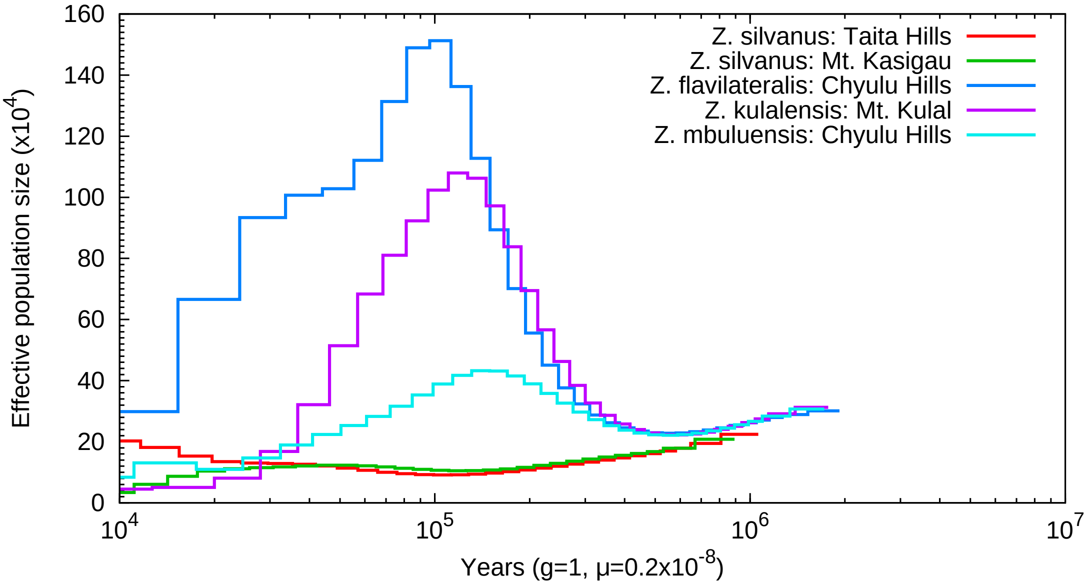

Figure S9: PSMC results of effective population size over time for highland and lowland Zosterops species across Kenya, including *Z. silvanus* in the fragmented Taita Hills forest (red) and the preserved Mt. Kasigau (green) as well as its lowland congener *Z. flavilateralis* (dark blue). Further sky-island species *Z. kulalensis* (purple) and *Z. mbuluensis* (light blue) shown for reference. Time scaling: Generation time = one year; Mutation rate per nucleotide = 0.2×10^{-8}.

congruent curve between *Z. silvanus* inhabiting the highly fragmented Taita Hills compared to the pristine Mt. Kasigau. The PSMC model suggests initial divergence of *Z. silvanus* inhabiting both the Taita Hills and Mt. Kasigau with low effective population size, while the lowland congener *Z. flavilateralis* experienced rapid growth. In the more recent past, we observed an increase in effective population size in *Z. silvanus* in the Taita Hills, while all other highland species contracted, despite the most restricted geographical range being in the Taita Hills. This increase in N_e could be possibly explained by a) range expansion or b) hybridization. While the former reason can be excluded given that the Taita Hills have been isolated for millions of years (i.e. there is no evidence that spread of the mountain cloud forest into the surrounding savannah habitat has occurred in the past), introgressive hybridization with another species might be the most plausible scenario. To this end, we expect introgressive hybridization, as a form of genetic rescue, from the lowland congener *Z. flavilateralis* into *Z. silvanus* inhabiting these small forest fragments, an assumption that forms the logical basis for the scenario building reported in this study.

References

Aben, J., Strubbe, D., Adriaensen, F., Palmer, S. C., Travis, J., Lens, L. & Matthysen, E. (2014). Simple individual-based models effectively represent Afrotropical forest bird movement in complex landscapes. https://doi.org/10.1111/1365-2664.12224

Adhikari, H., Heiskanen, J., Siljander, M., Maeda, E., Heikinheimo, V. & Pellikka, P. K. E. (2017). Determinants of Aboveground Biomass across an Afromontane Landscape Mosaic in Kenya. *Remote Sensing*, *9*(8), 827. https://doi.org/10.3390/rs9080827

Bird, J. P., Martin, R., Akçakaya, H. R., Gilroy, J., Burfield, I. J., Garnett, S. T., Symes, A., Taylor, J., Şekercioğlu, Ç. H. & Butchart, S. H. M. (2020). Generation lengths of the world's birds and their implications for extinction risk. *Conservation Biology*, *34*(5), 1252–1261. https://doi.org/10.1111/cobi.13486
_eprint: https://conbio.onlinelibrary.wiley.com/doi/pdf/10.1111/cobi.13486

Brown, J., Gillooly, J., Allen, A., Savage, V. & West, G. (2004). Toward a metabolic theory of ecology. *Ecology*, *85*(7), 1771–1789.

Githiru, M., Lens, L., Adriaensen, F., Mwang'ombe, J. & Matthysen, E. (2011). Using science to guide conservation: From landscape modelling to increased connectivity in the Taita Hills, SE Kenya. *Journal for Nature Conservation*, *19*(5), 263–268. https://doi.org/10.1016/j.jnc.2011.03.002

Grimm, V., Berger, U., DeAngelis, D. L., Polhill, J. G., Giske, J. & Railsback, S. F. (2010). The ODD protocol : A review and first update. *Ecological Modelling*, *221*, 2760–2768. https://doi.org/10.1016/j.ecolmodel.2010.08.019

Hole-filled seamless SRTM data V4. (2018). International Centre for Tropical Agriculture (CIAT). http://srtm.csi.cgiar.org

Jetz, W., Sekercioglu, C. H. & Böhning-Gaese, K. (2008). The Worldwide Variation in Avian Clutch Size across Species and Space. *PLOS Biology*, *6*(12), e303. https://doi.org/10.1371/journal.pbio.0060303

Leidinger, L., Vedder, D. & Cabral, J. S. (2021). Temporal environmental variation may impose differential selection on both genomic and ecological traits. *Oikos*, *130*(7), 1100–1115. https://doi.org/10.1111/oik.08172
_eprint: https://onlinelibrary.wiley.com/doi/pdf/10.1111/oik.08172

Leroy, T., Anselmetti, Y., Tilak, M.-K., Bérard, S., Csukonyi, L., Gabrielli, M., Scornavacca, C., Milá, B., Thébaud, C. & Nabholz, B. (2019). A bird's white-eye view on neosex chromosome evolution, 505610. https://doi.org/10.1101/505610

Li, H. & Durbin, R. (2009). Fast and accurate short read alignment with Burrows–Wheeler transform. *Bioinformatics*, *25*(14), 1754–1760. https://doi.org/10.1093/bioinformatics/btp324

Mulwa, R. K., Bennun, L. A., Ogol, C. K. P. O. & Lens, L. (2007). Population status and distribution of Taita White-eye Zosterops silvanus in the fragmented forests of Taita Hills and Mount Kasigau, Kenya. *Bird Conservation International*, *17*(2), 141–150. https://doi.org/10.1017/S0959270907000664

Nadachowska-Brzyska, K., Li, C., Smeds, L., Zhang, G. & Ellegren, H. (2015). Temporal Dynamics of Avian Populations during Pleistocene Revealed by Whole-Genome

Sequences. *Current Biology*, *25*(10), 1375–1380. https://doi.org/10.1016/j.cub.2015.03.047

Palmer, S. C. F., Coulon, A. & Travis, J. M. J. (2011). Introducing a 'stochastic movement simulator' for estimating habitat connectivity. *Methods in Ecology and Evolution*, *2*(3), 258–268. https://doi.org/10.1111/j.2041-210X.2010.00073.x _eprint: https://besjournals.onlinelibrary.wiley.com/doi/pdf/10.1111/j.2041-210X.2010.00073.x

Patton, A. H., Margres, M. J., Stahlke, A. R., Hendricks, S., Lewallen, K., Hamede, R. K., Ruiz-Aravena, M., Ryder, O., McCallum, H. I., Jones, M. E., Hohenlohe, P. A. & Storfer, A. (2019). Contemporary Demographic Reconstruction Methods Are Robust to Genome Assembly Quality: A Case Study in Tasmanian Devils. *Molecular Biology and Evolution*, *36*(12), 2906–2921. https://doi.org/10.1093/molbev/msz191

Pellikka, P. K. E., Clark, B. J., Gosa, A. G., Himberg, N., Hurskainen, P., Maeda, E., Mwang'ombe, J., Omoro, L. M. & Siljander, M. (2013). Agricultural Expansion and Its Consequences in the Taita Hills, Kenya. In *Developments in Earth Surface Processes* (pp. 165–179). Elsevier. https://doi.org/10.1016/B978-0-444-59559-1.00013-X

Pellikka, P. K. E., Heikinheimo, V., Hietanen, J., Schäfer, E., Siljander, M. & Heiskanen, J. (2018). Impact of land cover change on aboveground carbon stocks in Afromontane landscape in Kenya. *Applied Geography*, *94*, 178–189. https://doi.org/10.1016/j.apgeog.2018.03.017

QGIS.org. (2020). *QGIS Geographic Information System* (Version 3.14). https://www.qgis.org

Saito, M. & Matsumoto, M. (2008). SIMD-Oriented Fast Mersenne Twister: A 128-bit Pseudorandom Number Generator. In A. Keller, S. Heinrich & H. Niederreiter (Eds.), *Monte Carlo and Quasi-Monte Carlo Methods 2006* (pp. 607–622). Berlin, Heidelberg, Springer Berlin Heidelberg. https://doi.org/10.1007/978-3-540-74496-2_36

Schiffers, K., Schurr, F. M., Travis, J. M. J., Duputié, A., Eckhart, V. M., Lavergne, S., McInerny, G., Moore, K. A., Pearman, P. B., Thuiller, W., Wüest, R. O. & Holt, R. D. (2014). Landscape structure and genetic architecture jointly impact rates of niche evolution. *Ecography*, *37*(12), 1218–1229. https://doi.org/10.1111/ecog.00768

Smeds, L., Qvarnström, A. & Ellegren, H. (2016). Direct estimate of the rate of germline mutation in a bird. *Genome Research*, *26*(9), 1211–1218. https://doi.org/10.1101/gr.204669.116

Teucher, M., Schmitt, C. B., Wiese, A., Apfelbeck, B., Maghenda, M., Pellikka, P. K. E., Lens, L. & Habel, J. C. (2020). Behind the fog: Forest degradation despite logging bans in an East African cloud forest. *Global Ecology and Conservation*, *22*, e01024. https://doi.org/10.1016/j.gecco.2020.e01024

Uecker, H., Setter, D. & Hermisson, J. (2015). Adaptive gene introgression after secondary contact. *Journal of Mathematical Biology*, *70*(7), 1523–1580. https://doi.org/10.1007/s00285-014-0802-y

Wagura, L. (2018). *Forest Fragments Connectivity to Save Two Critically Endangered endemic birds from possible extinction in the Taita Hills.* African Bird Club. Nairobi.
